# Novel Antibacterial 4-Piperazinylquinoline Hybrid Derivatives Against *Staphylococcus aureus*: Design, Synthesis, and In Vitro and In Silico Insights

**DOI:** 10.3390/molecules30010028

**Published:** 2024-12-25

**Authors:** Gabriele La Monica, Annamaria Gallo, Alessia Bono, Federica Alamia, Antonino Lauria, Rosa Alduina, Annamaria Martorana

**Affiliations:** 1 Department of Biological, Chemical and Pharmaceutical Sciences and Technologies (STEBICEF), University of Palermo, Viale delle Scienze, 90128 Palermo, Italy; gabriele.lamonica01@unipa.it (G.L.M.); annamaria.gallo01@unipa.it (A.G.); alessia.bono01@unipa.it (A.B.); federica.alamia01@unipa.it (F.A.); antonino.lauria@unipa.it (A.L.); valeria.alduina@unipa.it (R.A.); 2 NBFC, National Biodiversity Future Center, Piazza Marina 61, 90133 Palermo, Italy

**Keywords:** molecular hybridization, quinoline, piperazine, *Staphylococcus aureus*, antibiotic activity, heterocyclic compounds, structure-based studies

## Abstract

Molecular hybridization, which consists of the combination of two or more pharmacophores into a single molecule, is an innovative approach in drug design to afford new chemical entities with enhanced biological activity. In the present study, this strategy was pursued to develop a new series of 6,7-dimethoxy-4-piperazinylquinoline-3-carbonitrile derivatives (**5a**–**k**) with potential antibiotic activity by combining the quinoline, the piperazinyl, and the benzoylamino moieties, three recurrent frameworks in antimicrobial research. Initial in silico evaluations were conducted on the designed compounds, highlighting favorable ADMET and drug-likeness properties, which were synthesized through a multistep strategy, isolated, and fully characterized. The whole set was tested in vitro against *Staphylococcus aureus* ATCC 25923 and *Pseudomonas aeruginosa* ATCC 10145 representative Gram-positive and Gram-negative strains, respectively. Notably, **5k** exhibited potent and selective activity against *S. aureus* (MIC 10 μM), with a dose- and time-dependent response and capability to affect cell membrane integrity. On the other hand, no significant activity was observed against *P. aeruginosa*. Further in silico docking and molecular dynamics studies highlighted strong interactions of **5k** with bacterial enzymes, such as tyrosyl-tRNA synthetase, pyruvate kinase, and DNA gyrase B, suggesting potential modes of action. These findings underscore the value of the hybridization approach in producing new antimicrobial agents, guiding future optimization for broader-spectrum activity.

## 1. Introduction

In the search for new small molecules to treat infectious diseases, nitrogen heterocycles are consistently prominent and successful scaffolds, frequently serving as essential pharmacophores in various approved antimicrobial drugs [1,2]. Of particular interest are bicyclic nitrogen-containing structures, such as quinolone and quinoline cores, which are widely represented in fluoroquinolone antibiotics (e.g., ciprofloxacin and its derivatives), one of the most prescribed classes of broad-spectrum antibiotics [3,4,5].

Another important nitrogen heterocycle in antimicrobial research is the six-membered alicyclic piperazine ring. Its unique chemical properties, including a large polar surface area, relative structural rigidity, and hydrogen bond donor/acceptor groups, make it an effective contributor to enhancing water solubility, oral bioavailability, ADMET (absorption, distribution, metabolism, excretion, and toxicity) properties, and target affinity and specificity in compounds [6,7,8].

Taking this into account, such a valuable and well-exploited approach in the medicinal chemistry field to develop new more bioactive small molecules is molecular hybridization, i.e., the combination of two or more pharmacophores into a single chemical entity. The integration of two bioactive frameworks could lead to new compounds with both improved activity and drug-likeness properties, combining the beneficial aspects of all components [9,10]. In particular, quinoline hybrids have shown great potential in antimicrobial research, with quinoline-piperazine hybrids attracting attention due to their activity against both Gram-positive and Gram-negative bacteria as well as various mycobacteria [11,12,13,14,15,16].

**Figure 1:**
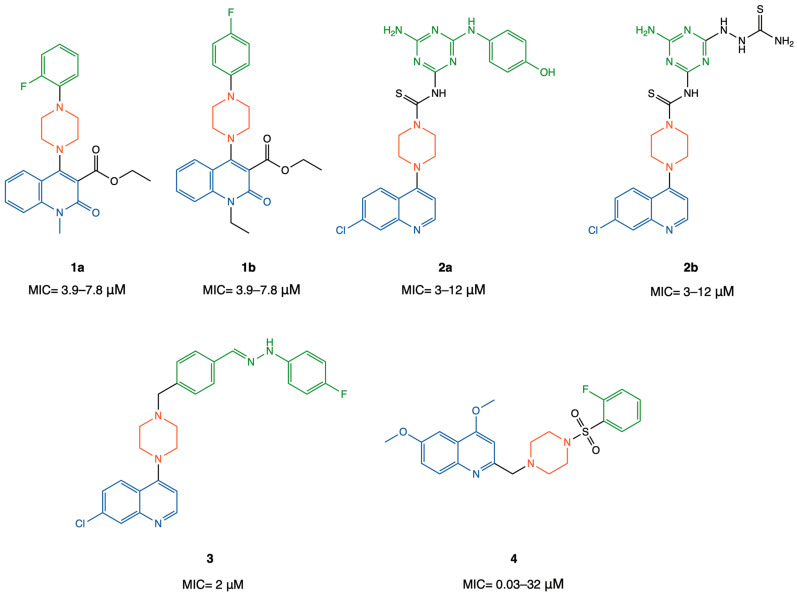
Chemical structure of antibacterial piperazinyl-quinoline hybrid derivatives reported in the literature, with the corresponding MIC (Minimum Inhibitory Concentration) ranges (details on the susceptible bacterial strains are reported in the text and in the corresponding references).

Figure 1 shows several notable quinoline-piperazine hybrids with antimicrobial properties. Most of these compounds feature a piperazine ring attached to the C4 position of the quinoline ring (e.g., compounds **1**–**3**), although recent studies have also investigated the C2 attachment (compound **4**).

In detail, compounds **1a**,**b** showed promising activity against both Gram-positive and negative bacterial strains, with MIC values in the range of 3.9–7.8 μM on *Staphylococcus aureus*, *Pseudomonas aeruginosa*, *Bacillus subtilis*, and *Escherichia coli* [17]. Also, 4-piperazinylquinoline compounds **2a**,**b**, with a hydrophobic side portion of 1,3,5-triazine, showed noteworthy MIC values in the range of 3–12 μM against *S. aureus*, *P. aeruginosa*, and *E. coli* [18]. Compound **3**, with an uncommon hydrazone moiety, showed an MIC of 2 μM against *S. aureus* [19]. On the other hand, in very recent times, some 2-[(piperazin-1-yl)methyl]quinoline showed remarkable antibiotic activity, with compound **4** exhibiting an MIC in the range of 0.03–32 μM against a panel of Gram-positive bacteria: *S. aureus, S. epidermidis, Enterococcus faecalis,* and *Enterococcus faecium* [20].

From a structural perspective, these quinoline-piperazine hybrids incorporate a hydrophobic aromatic side chain (indicated in green), which, together with the core pharmacophoric framework, enhances the desired biological effect. This structural synergy offers a promising pathway for developing more potent antimicrobial agents.

In this study, a new series of potential antimicrobial hybrid quinoline-piperazine is proposed. The newly designed derivatives were firstly evaluated in silico for their potential ADMET and drug-likeness properties, then synthetized through appropriate synthetic procedures and fully characterized. The entire set was then evaluated in vitro against two representative bacteria, *Pseudomonas aeruginosa* and *Staphylococcus aureus*, belonging to Gram-negative and Gram-positive classes, respectively. Molecular modeling studies were also performed to suggest a potential mechanism of action for the most promising compounds.

## 2. Results and Discussion

### 2.1. In-House Database Design and Physicochemical, Drug-Likeness, and ADME Properties Prediction

**Figure 2:**
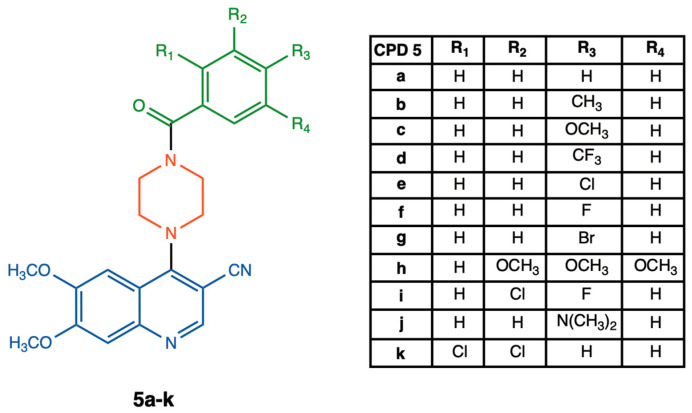
General chemical structure of the in-house database of 6,7-dimethoxy-4-piperazinylquinoline-3-carbonitrile derivatives **5a**–**k** explored in this work.

In light of the promising potential of quinoline-piperazine hybrids in antimicrobial research, and considering our experience in the design and synthesis of bioactive quinoline compounds [21,22,23,24], this study introduces a new in-house database of 4-piperazinylquinoline derivatives (compounds **5a**–**k**, Figure 2), designed with reference to the general skeleton described in the introduction for this class of compounds (Figure 1). Specifically, as shown in Figure 2, three distinct pharmacophoric regions were merged to create a unique molecular hybrid where the piperazine core acts as a bridge between the 6,7-dimethoxyquinoline-3-carbonitrile scaffold and a benzoyl moiety. The benzoyl fragment is variously substituted with alkyl, alkoxy, halogen, and alkylamino groups.

Given the high failure rates in drug discovery due to physicochemical and pharmacokinetic/toxicokinetic challenges, early-stage assessment through in silico techniques is essential [25,26,27,28]. For these reasons, the initial part of the study involved a preliminary assessment of ADMET and drug-likeness properties for the entire database. To this aim, SwissADME [29] and QikProp [30] were employed, being the most reliable tools for predicting a wide range of parameters related to all pharmacokinetic phases, including absorption, distribution, metabolism, excretion, and toxicity (the full matrices related to all the parameters computed by each tool are provided in the Supporting Information S1).

In general, as shown in Table 1, all the proposed compounds (**5a**–**k**) exhibited favorable ADME properties, with all predicted parameters falling within optimal ranges: molecular weight values of 150–500 g/mol; a balanced lipophilicity/hydrophilicity profile with an average LogP value of 3 and LogS value of −4.7; total polar surface area values between 20 Å and 130 Å; and an ideal ratio of saturated to unsaturated bonds (fraction of Csp3 and rotatable bonds). Additionally, all derivatives, particularly **5k**, were predicted to be highly permeable across biological membranes, as demonstrated by the excellent predicted permeability across the Caco-2 membrane (QPPCaco > 500), a well-established model for assessing passive transport across biological membranes.

**Figure 3:**
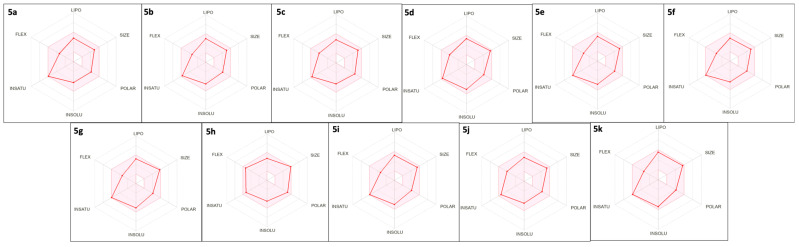
Bioavailability radar plot generated by the SwissADME tool for compounds **5a**–**k**, which graphically illustrates the optimal range (pink area) for lipophilicity (−0.7 < LogP < +5), solubility (−6 < LogS < 0), size (150 g/mol < MW < 500 g/mol), flexibility (0 < RB < 9), polarity (20 Å < TPSA < 130 Å), and unsaturation (0.25 < fraction of Csp^3^ < 1) required for an orally bioavailable drug.

**Figure 4:**
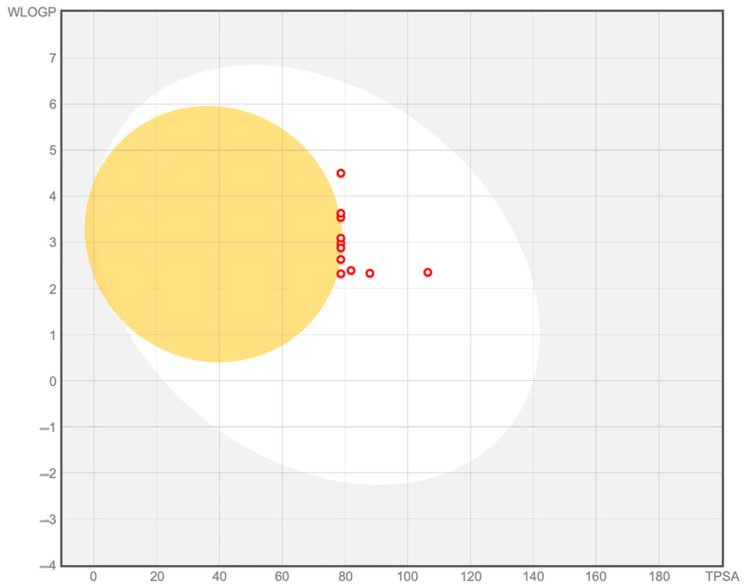
BOILED-Egg plot generated by the SwissADME tool for compounds **5a**–**k**. Quinoline derivatives in the grey region are predicted to lack passive absorption in the gastrointestinal tract (indicating low oral bioavailability), while those in the white region are expected to be passively absorbed in the intestine. Compounds in the yellow (yolk) region are anticipated to cross the blood–brain barrier (BBB). Red dots represent compounds that are not substrates of P-gp.

Thus, the entire set met the required criteria for oral bioavailability—a highly feasible route of administration—crucial for antibiotics intended to treat systemic infections. As an additional tool for visualizing bioavailability potential, Figure 3 and Figure 4 are provided, showing the bioavailability radar and the BOILED-Egg plots generated by the SwissADME tool, respectively [29,31]. Specifically, the first graph is a hexagonal plot, where each vertex represents a distinct ADME property listed in Table 1, and the pink area indicates the optimal range for oral absorption. As shown, the perimeter for all compounds lies entirely within this optimal region.

This was also confirmed by the BOILED-Egg plot in Figure 4, which, through the correlation between the LogP and the TPSA values, suggests a model to predict gastrointestinal (GI) absorption and blood–brain barrier (BBB) permeation as well as possible active transport phenomena mediated by P-glycoprotein responsible for altered absorption, distribution, and excretion. The entire set of compounds falls within the white region, and in some cases, along the border between yellow and white, indicating their capability to cross the GI tract. Additionally, as shown by the red dots, none of the compounds were predicted to be substrates of glycoprotein P (P-gp), a well-characterized efflux pump that often contributes to suboptimal bioavailability.

Additionally, the dataset was evaluated for drug-likeness and medicinal chemistry compatibility. Table 2 lists several commonly used rules and filters in virtual screening campaigns to exclude structures with potential issues early in the research process. The entire set displayed optimal drug-likeness properties with no significative violations of key rules (Lipinski [32], Ghose [33], Veber [34], Egan [35], Muegge [36]). Moreover, they presented no medicinal chemistry alerts, showing an absence of promiscuous fragments (PAINS, Pan-Assay Interference Compounds [37]) and toxicophores (Brenk alerts [38]).

### 2.2. Synthesis of 4-(4-Benzoylpiperazin-1-yl)-6,7-dimethoxyquinoline-3-carbonitrile Derivatives ***5a**–**k***

**Scheme 1:**
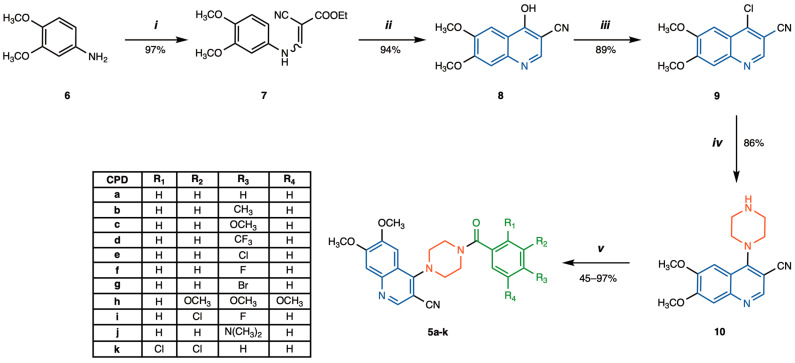
Synthesis of 6,7-dimethoxy-4-piperazinylquinoline derivatives **5a**–**k**. Reagents and conditions: (i) ethyl 2-cyano-3-ethoxyacrylate, toluene, reflux, 2 h; (ii) Dowtherm A, N_2_, reflux, 10 h; (iii) POCl_3_, reflux, 4 h; (iv) anhydrous piperazine (2.5 eq.), anhydrous MeCN, reflux, 2 h (v) appropriate benzoyl chloride (1.25 eq.), DIPEA (1.5 eq.), anhydrous DCM, rt, 24 h.

The favorable ADMET predictions for the 4-(4-benzoylpiperazin-1-yl)-6,7-dimethoxyquinoline-3-carbonitrile derivatives **5a**–**k** prompted us to synthesize them. Scheme 1 illustrates the preparation process, which involved five preparative steps. In detail, the quinoline scaffold was built up in the first three reactions (i–iii, intermediates **6**–**9**), followed by coupling with a piperazine fragment to give the 4-piperazinylquinoline hybrid intermediate **10** (step iv). Finally, the introduction of the aromatic side chain was introduced by reacting the quinoline-piperazine derivative with various benzoyl chlorides (step v) decorated with suitable substituents (e.g., alkyl, alkoxy, halogen, alkylamino groups), affording the title **5a**–**k** derivatives.

For the synthesis of the quinoline core, the well-established Gould–Jacobs methodology was applied owing to its reliability in producing a key 4-hydroxyquinoline intermediate of type **8**. In general, it consists of the preliminary synthesis of an anilino-methylene malonate intermediate, which is then cyclized under forcing conditions [39].

Specifically, 3,4-dimethoxyaniline (compound **6**) was selected as the starting material and was reacted with ethyl 2-cyano-3-ethoxyacrylate, yielding intermediate **7** as a mixture of the two E/Z regioisomers in excellent yields (two different spots in the TLC) within a relatively short reaction time (2 h). The subsequent intramolecular cyclization of this intermediate led to the formation of 4-hydroxy-6,7-dimethoxyquinoline-3-carbonitrile (compound **8**) with an optimal 94% yield. The cyclization was achieved by prolonged heating at 257–260 °C (10–12 h) using high-boiling solvent Dowtherm A. Aromatic chlorination with POCl_3_ then produced compound **9**, which possessed an optimal leaving group at the C4 position. The data obtained from this sequence of reactions were consistent with the literature [40].

After the synthesis of the desired dimethoxy-quinoline scaffold, the piperazine linker was introduced at the C4 position by nucleophilic aromatic substitution (SnAr). The activating effect of the electron-withdrawing nitrile group adjacent to the 4-Cl was partly mitigated by the presence of the two methoxy electron-donating group. As a result, heating the 4-chloroquinoline intermediate at reflux for 2 h with an excess of piperazine (2.5 eq.) was required to afford the desired intermediate **10** in high yields.

In the final stage, the aromatic side chain was added through a nucleophilic acyl substitution involving the second nitrogen atom of the piperazine with variously substituted benzoyl chlorides. This reaction was carried out under strictly anhydrous conditions using dry dichloromethane (DCM) as a solvent and *N*,*N*-diisopropylethylamine (DIPEA) as a base, resulting in the formation of 4-(4-benzoylpiperazin-1-yl)-6,7-dimethoxyquinoline-3-carbonitrile derivatives **5a**–**k**, which were obtained in moderate to excellent yields (45–97%). All isolated compounds were fully characterized spectroscopically (see Section 3.1), confirming the presence of the three fragments.

In this regard, the analysis of both ^1^H and ^13^CNMR spectra for the piperazine portion is particularly noteworthy and deserves special attention. As shown in the Section 3.1.2 and Supporting Information S4, the precursor compound **10** displays two distinct sharp triplets in ^1^H (3–3.5 ppm) and two distinct CH_2_ signals in ^13^CNMR (56.0–56.5 ppm, negative in the DEPT experiment), indicating two pairs of non-equivalent CH_2_ groups, each in a distinct chemical environment. Nevertheless, such a pattern is not found in the final compounds, where a sharp triplet and a very broad singlet (around 3.5–4 ppm) were often observed in the ^1^HNMR. In addition, only a single signal was observed for all CH_2_ groups in the ^13^CNMR (around 52.0 ppm). This change likely arose from the high mobility of piperazinyl methylene protons within the flexible, aliphatic ring as well as the similar electronic characteristics of the quinoline and benzoyl groups, which lead to a convergence and broadening of signals.

An exception was **5k**, which contains a 2,3-dichlorobenzoyl group. In this case, the CH_2_ protons exhibited distinct signals in both ^1^H and ^13^CNMR, suggesting a more rigid and locked conformation in 3D space. This conformational rigidity rendered the piperazine protons non-equivalent, distinguishing compound **5k** from the other derivatives.

All the piperazinyl quinolines **5a**–**k** were then isolated with optimal purity (>95%) and evaluated in vitro, as described in the following section.

### 2.3. Biological Activity

To thoroughly analyze the antimicrobial potential of the novel 4-(4-benzoylpiperazin-1-yl)-6,7-dimethoxyquinoline-3-carbonitrile derivatives (**5a**–**k**), in vitro assays were conducted against two reference bacterial strains, *Staphylococcus aureus* ATCC 25923 and *Pseudomonas aeruginosa* ATCC 10145, which are representative of the Gram-positive and Gram-negative bacterial classes, respectively. *S. aureus* ATCC 25923 is a clinical isolate with resistance to multiple antibiotics [41], while *P. aeruginosa* ATCC 10145 is widely used as a standard control strain in laboratory drug testing. The intermediate **10** was tested for comparison to determinate how the introduction of the benzoyl portions affected the biological activity.

**Figure 5:**
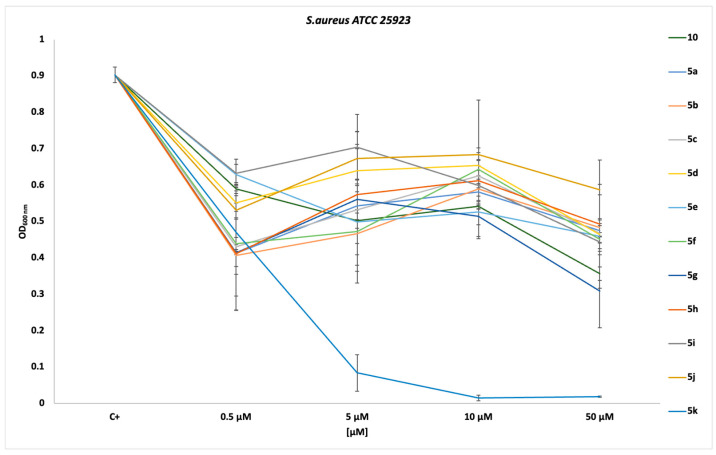
MIC of compounds **10** and **5a**–**k** against the reference Gram-positive pathogen *S. aureus* ATCC 25923. The data are the mean ± SD derived from a minimum of three independent experiments.

In terms of antibiotic activity against *S. aureus*, Figure 5 shows the Minimal Inhibitory Concentration (MIC) values (indicating bacteriostatic effect) of the tested piperazinyl quinolines, ranging from 0.5 to 50 μM. Among these, **5k** was the only one demonstrating significant antibacterial activity against the Gram-positive reference strain (Figure 5), with an MIC of 10 μM. No other derivatives showed an appreciable effect on *S. aureus* growth. This unique activity of compound **5k** could be attributed to both its physicochemical and structural properties. As discussed in previous sections, the presence of two chlorine atoms on the benzoyl fragment contributes to higher lipophilicity (logP of 3.55) and enhanced predicted permeability across biological membranes as well as a distinct three-dimensional conformation. These characteristics may facilitate improved membrane penetration in Gram-positives, such as *S. aureus*, and more favorable interactions with biological targets.

However, the Minimum Bactericidal Concentration (MBC) value could not be determined. Even at the highest tested concentration of 250 µM, **5k** showed no bactericidal activity, but a bacteriostatic effect, as evidenced by a 4-log_10_ reduction in CFU/mL compared to the untreated cultures (Supplementary Figure S2).

**Figure 6:**
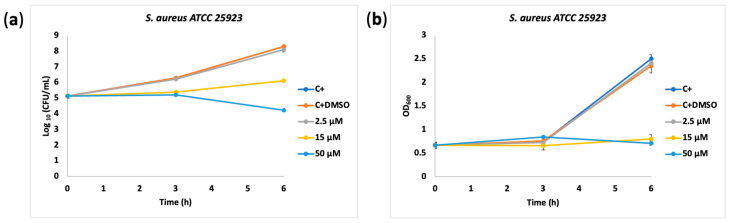
Dose- and time-dependent effects of **5k** against *S. aureus* ATCC 25923 cell viability after 3 and 6 h of treatment: (**a**) Minimal Inhibitory Concentration of the **5k** against the reference pathogen *S. aureus* ATCC 25923; (**b**) Minimal Bactericidal Concentration of the **5k** dose-dependent experiment against *S. aureus* ATCC 25923. C+ indicates *S. aureus* ATCC 25923 cells without treatment, and C+DMSO indicates *S. aureus* ATCC 25923 cells treated with DMSO. The data are the mean ± SD derived from a minimum of three independent experiments.

In light of these preliminary results, further investigations were conducted on compound **5k** to analyze dose- and time-dependent effects. Three concentrations (2.5, 15, and 50 μM) were selected to explore the time-dependent effect of the molecule. After 3 h, the number of cells was the same for all concentrations tested and for the untreated sample. After 6 h, a different trend emerged: the 15 and 50 μM treatments prevented any growth, while the 2.5 μM treated cells displayed the same exponential growth as the control and the DMSO-treated cells (Figure 6a). A similar effect was observed on the viable count with treatments at 15 and 50 μM, significantly enhancing the effect at 50 μM.

**Figure 7:**
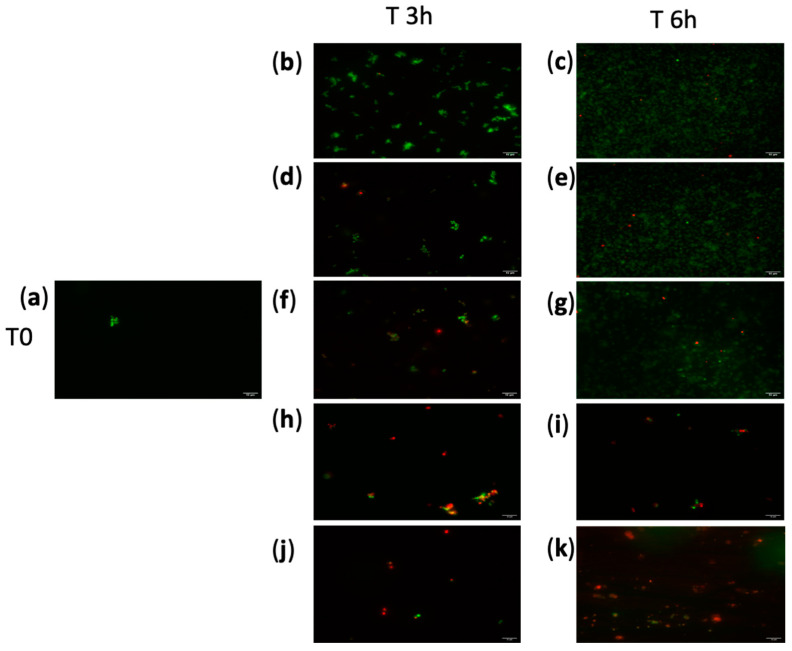
Fluorescence microscopy images of *S. aureus* ATCC 25923 cells were taken at the start of the treatment (T0) and after 3 and 6 h in the absence or presence of 2.5 μM, 15 μM, and 50 μM of compound **5k**. (**a**) *S. aureus* ATCC 25923 cells at the initial treatment point (T0); (**b**,**c**) *S. aureus* ATCC 25923 cells untreated after 3 h (h) and 6 h, respectively; (**d**,**e**) *S. aureus* ATCC 25923 cells treated with DMSO 100% after 3 h and 6 h, respectively; (**f**,**g**) *S. aureus* ATCC 25923 cells treated with 2.5 μM of **5k** after 3 h and 6 h, respectively; (**h**,**i**) *S. aureus* ATCC 25923 cells treated with 15 μM of **5k** after 3 h and 6 h, respectively; (**j**,**k**) S. aureus ATCC 25923 cells treated with 50 μM of **5k** after 3 h and 6 h. Magnification 63×. The green cells are viable, the red cells are dead, and the orange cells are viable with damaged membranes. Scale bar 10 µm.

Fluorescence images were then taken to gain insight into the mechanism of action of 4-piperazinylquinoline **5k** on *S. aureus*. In accordance with growth profiles, the fluorescence microscopy images showed a strong green fluorescent signal exclusively in the unchallenged samples and in the 2.5 μM challenged cells after 3 h and 6 h. In contrast, the cells treated with 15 μM resulted in a mixed population of viable and non-viable cells, evidenced by simultaneous green and red fluorescence, and finally, the cells treated with 50 μM showed a preponderant red fluorescence. These results indicate that the compound compromised cell membrane integrity since the propidium iodide (red dye) can penetrate only into the damaged cells (Figure 7a–k).

To complete the preliminary antibacterial analysis, the entire series of 4-piperazinylquinolines **5a**–**k** were tested against the Gram-negative *P. aeruginosa* ATCC 10145. None of the compounds tested showed activity, even at the highest concentration used. This result could be due to the different membrane composition between Gram-negative and Gram-positive bacterial strains. Indeed, it is known that Gram-negative bacteria possess an additional outer membrane composed of lipoproteins and lipopolysaccharides (LPS) that is more difficult to cross since it acts as a hydrophobic barrier that restricts molecule entry into the cells [42,43,44]. The presence of the LPS layer in Gram-negative bacteria significantly enhances their resistance, making infections more challenging to eliminate [44].

Furthermore, all derivatives of type **5** were tested for hemolytic activity on blood agar. The most active compound **5k** showed no hemolytic activity, underscoring its safety profile.

### 2.4. In Silico Insights into the Mechanism of Action of ***5k*** on S. aureus: Induced Fit Docking and Molecular Dynamic Simulation Studies

Following the promising in vitro results obtained for the dichloro compound **5k** against *S. aureus* ATCC 25923 strains, further in silico analyses were conducted to explore its specific and selective antibiotic activity against this Gram-positive bacterium. To the best of our knowledge, only a few studies have investigated the mechanism of action of piperazinyl quinoline compounds with antibiotic activity through molecular modeling techniques, as for example, compounds **1a,b** (see ref. [17]).

A thorough literature search highlighted several *S. aureus*-specific proteins, integral to its virulence and spread, as potential targets for small antimicrobial molecules. Selected targets included proteins involved in nucleotide biosynthesis and DNA replication (e.g., DNA gyrase, thymidylate kinase, dihydrofolate reductase) [45,46,47], enzymes essential for protein biosynthesis (e.g., aminoacyl-tRNA synthetases) [48], enzymes in critical metabolic pathways for ATP production and cell structure synthesis as pyruvate kinase, C30 carotenoid dehydrosqualene synthase (CrtM) [49,50], and those involved in cell division (e.g., FtsZ) [51].

Based on these initial findings, structure-based Induced Fit Docking (IFD) studies were conducted to evaluate the binding affinity of compound **5k** against seven selected *S. aureus* protein targets, each with a resolved crystal structure available in the Protein Data Bank. Table 3 presents the computed parameters (IFD, glide, and prime energy scores) for the optimal docking pose of **5k** against each target, along with comparison scores for a set of known reference inhibitors. The complete set of docking results is available in the Supporting Information.

**Figure 8:**
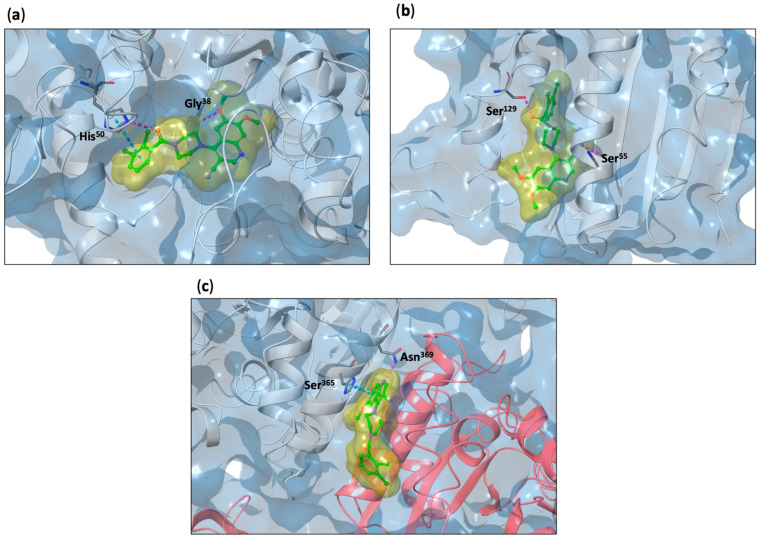
3D representation (surface view) of the best docked poses of **5k** in complex with TyrRS (**a**), gyrase B (**b**) and homodimeric pyruvate kinase (**c**). Interactions with the most important amino acids are highlighted with colored dots. Protein secondary structures, helices and sheets, are evidenced.

The findings indicated that the tested compound demonstrates moderate to strong binding affinity for tyrosyl-tRNA synthetase, pyruvate kinase, and DNA gyrase B, comparable to known reference inhibitors. This was validated by IFD, glide, and prime energy scores that were either higher or within similar ranges. Figure 8a–c presents the 3D structure of the best docked poses of **5k** in complex with the top-ranked targets. A visual analysis revealed that the quinoline-based compound effectively penetrated the three binding pockets, establishing multiple interactions with essential amino acid residues and adopting an orientation similar to that of the co-crystallized reference ligands (see Supporting Information).

On the other hand, a weaker interaction was predicted for the rest of the investigated proteins, with IFD values lower than the reference inhibitors.

To further assess the binding stability with these three enzymes over time, an all-atom molecular dynamics (MD) simulation was conducted on the top docked poses of **5k** in complex with each target over a 100 ns timeframe.

To evaluate the stability of the ligand-protein complexes, the simulation trajectories were initially visually inspected, confirming that all complexes retained stability throughout the simulation, showing no significant structural distortions, large domain shifts in the proteins, or ligand displacement from binding sites.

Subsequently, a detailed analysis was conducted by calculating the root mean square deviation (RMSD), a key geometric property which registers the displacement of selected atoms compared to the starting structure (t = 0). A stable average RMSD with minimal fluctuations (typically between 1–3 Å) indicates system stability and proper equilibration, while larger fluctuations may suggest structural changes or incomplete equilibration.

**Figure 9:**
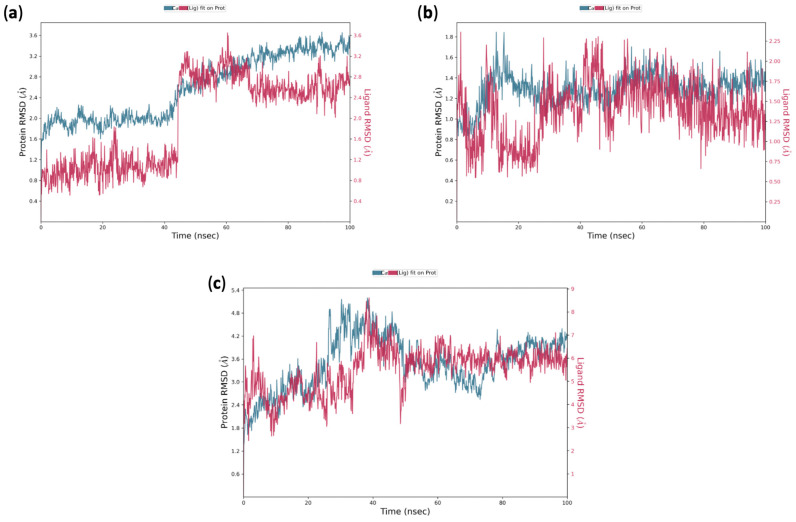
Plots illustrating the root mean square deviation (RMSD) of the protein backbone and ligand (in Å) for the complexes of **5k** with TyrRS (**a**), gyrase B (**b**), and pyruvate kinase (**c**) over a duration of 100 ns. Each plot features dual Y-axes: the left Y-axis represents the RMSD for the protein backbone (Cα atoms), while the right Y-axis indicates the RMSD for the heavy atoms of the ligand. The X-axis denotes the simulation duration in nanoseconds. The blue lines depict the RMSD values for the protein backbone, whereas the red lines reflect the RMSD of the ligand-heavy atoms aligned with the protein backbone.

In Figure 9a–c, the RMSD values for the protein backbone (Cα atoms) and the heavy atoms of the ligand are presented throughout the entire simulation period. Overall, all three complexes exhibited acceptable RMSD values for both the ligand (**5k**) and the protein backbones, showing only slight fluctuations after 40–50 ns of simulation. For comparison, the plots corresponding to the reference inhibitors are included in the Supporting Information. Notably, a higher RMSD value was observed for the ligand when bound to pyruvate kinase (Figure 9c). This observation may be attributed to the unique characteristics of the protein’s binding site, which is located on the surface between two monomers rather than in a deep, confined pocket.

In conclusion, this analysis further supports the moderate to high stability of the investigated ligand-protein complexes, reinforcing the notion that these targets may play a significant role in the mechanism of antibiotic activity of **5k** against *S. aureus*.

## 3. Materials and Methods

### 3.1. Chemistry

#### 3.1.1. General Information

Unless otherwise specified, all reagents and solvents were obtained from commercial suppliers Merck (Darmstadt, Germany), VWR International (Milan, Italy), Alfa Aesar (Haverhill, MA, USA), and Acros Organics (Segrate, Italy) and used without additional purification. Melting points were measured on a Büchi Tottoli capillary apparatus and were uncorrected (Büchi, Cornaredo, Italy). The ^1^HNMR and ^13^CNMR spectra were acquired at 400 MHz and 100 MHz, respectively, in CDCl_3_ or DMSO-d_6_ using a Bruker AC-E series 400 MHz spectrometer (Bruker, Milan, Italy). Chemical shift values are reported in parts per million (ppm) with tetramethylsilane (TMS) as the internal standard. The following abbreviations are used: br s (broad signal), s (singlet), d (doublet), t (triplet), q (quartet), m (multiplet), and rt (room temperature). The purity of all compounds tested in biological assays was confirmed to be greater than 95% by high-performance liquid chromatography/mass spectrometry (HPLC/MS) analysis. Mass spectrometry was performed using an Agilent (Santa Clara, CA, USA) 6540 UHD accurate-mass quadrupole time-of-flight (Q-TOF) spectrometer. Microanalytical data were consistent with theoretical values within ±0.4%. Thin-layer chromatography (TLC) was conducted on precoated silica gel GF254 plates, and compounds were visualized using a UV lamp at 254 nm. Column chromatography was carried out using Merck silica gel (230 and 400 mesh) or a Biotage (Uppsala, Sweden) FLASH40i chromatography system with prepacked cartridges. 3,4-dimethoxyaniline (**6**) is commercially available. The intermediates **7**–**9** were obtained following procedures reported in the literature [40,52]

#### 3.1.2. Experimental Procedures and Characterization of 6,7-Dimethoxy-4-piperazinylquinoline Derivatives

6,7-dimethoxy-4-(piperazin-1-yl)quinoline-3-carbonitrile (**10**)

To a stirring mixture of 4-chloro-6,7-dimethoxy-3-quinolinecarbonitrile **9** (400 mg, 1.61 mmol) in acetonitrile, piperazine (346 mg, 4.02 mmol) was added, and the mixture was heated at reflux for 2 h. Then, the solvent was evaporated in vacuo, and the crude was purified by chromatography column using DCM/MeOH 98:2 as an eluant. The yield was 86%; Mp 160–161 °C; ^1^HNMR (CDCl_3_) δ: 3.13–3.19 (m, 4H, 2 × CH_2_), 3.56–3.62 (m, 4H, 2 × CH_2_), 4.01 (s, 3H, OCH_3_), 4.03 (s, 3H, OCH_3_), 7.28 (s, 1H, H-5), 7.36 (s, 1H, H-8), 8.61 (s, 1H, H-2); ^13^CNMR (CDCl_3_) δ: 46.7 (CH_2_), 53.5 (CH_2_), 56.0 (CH_3_), 56.3 (CH_3_), 95.8, 102.5 (CH), 109.0 (CH), 118.3, 118.8, 147.8, 149.7, 151.1 (CH), 153.9, 158.1; HRMS-ESI [(M + H)^+^] *m*/*z* calculated for C_16_H_18_N_4_O_2_: 299.1503, found: 299.1501.

General procedure for the synthesis of 4-(4-benzoylpiperazin-1-yl)-6,7-dimethoxyquinoline-3-carbonitrile **5a**–**k**

To a stirred solution of **10** (100 mg, 0.33 mmol) in dry DCM, the DIPEA (0.5 mmol) was added at room temperature. The resultant mixture was cooled at 0 °C, and benzoyl chloride (0.41 mmol) was slowly added. The reaction mixture was stirred at room temperature (25 °C) for 24 h, and then the crude was washed with HCl_aq_ 1N and extracted with DCM. The organic layer was dried over sodium sulphate and evaporated under a vacuum. The residue was purified by column chromatography using EtOAc/Petroleum Ether (2:1) as an eluent. The product was recrystallized from Et_2_O.

4-(4-benzoylpiperazin-1-yl)-6,7-dimethoxyquinoline-3-carbonitrile (**5a**)

The yield was 87% (115 mg); Mp 208–209 °C; ^1^HNMR (CDCl_3_) δ: 3.64 (t, J = 5.0 Hz, 4H, 2 × CH_2_), 3.69–4.08 (br, 4H, 2 × CH_2_), 4.02 (s, 3H, OCH_3_), 4.05 (s, 3H, OCH_3_), 7.26 (s, 1H, H-5), 7.40 (s, 1H, H-8), 7.47 (m, 5H, J = 2.6 Hz, H-2′, H-3′, H-4′, H-5′, H-6′), 8.66 (s, 1H, H-1); ^13^CNMR (CDCl_3_) δ: 52.0 (CH_2_), 56.1 (CH_3_), 56.4 (CH_3_), 96.6, 101.9 (CH), 109.2 (CH), 118.3, 118.5, 127.2 (CH), 128.7 (CH), 130.2 (CH), 135.1, 148.0, 150.2, 150.8 (CH), 154.3, 157.3, 170.9; HRMS-ESI [(M + H)^+^] *m*/*z* calculated for C_23_H_22_N_4_O_3_: 403.1765, found: 403.1765.

6,7-dimethoxy-4-(4-(4-methylbenzoyl)piperazin-1-yl)quinoline-3-carbonitrile (**5b**)

The yield was 75% (103 mg); Mp 198–199 °C; ^1^HNMR (DMSO-d_6_) δ: 2.35 (s, 3H, CH_3_), 3.59 (br s, 8H, 4 × CH_2_), 3.95 (m, 6H, 2 × CH_3_), 7.26–7.38 (m, 6H, H-5, H-8, H-2′, H-3′, H-5′, H-6′), 8.64 (s, 1H, H-2). ^13^CNMR (DMSO-d_6_) δ: 21.4 (CH_3_), 52.1 (CH_2_), 56.2 (CH_3_), 56.5 (CH_3_), 95.6, 103.3 (CH), 109.1 (CH), 117.8, 119.1, 127.6 (CH), 129.5 (CH), 133.3, 139.8, 147.7, 150.0, 150.8 (CH), 154.3, 157.5, 170.1; HRMS-ESI [(M + H)^+^] *m*/*z* calculated for C_24_H_24_N_4_O_3_: 417.1921, found: 417.1920.

6,7-dimethoxy-4-(4-(4-methoxybenzoyl)piperazin-1-yl)quinoline-3-carbonitrile (**5c**)

The yield was 91% (130 mg); Mp 171–172 °C; ^1^HNMR (CDCl_3_) δ: 3.65 (t, 4H, J = 4.9 Hz, 2 × CH_2_), 3.86 (s, 3H, OCH_3_), 3.82–4.02 (m, 4H, 2 × CH_2_), 4.02 (s, 3H, OCH_3_), 4.05 (s, 3H, OCH_3_), 6.96 (d, 2H, J = 8.8 Hz, H-3′, H-5′), 7.26 (s, 1H, H-5), 7.40 (s, 1H, H-8), 7.47 (d, 2H, J = 8.8 Hz, H-2′, H-6′), 8.66 (s, 1H, H-2); ^13^CNMR (CDCl_3_) δ: 52.0 (CH_2_), 55.4 (CH_3_), 56.1 (CH_3_), 56.4 (CH_3_), 96.5, 101.9 (CH), 109.1 (CH), 113.9 (CH), 118.3, 118.5, 127.1, 129.4 (CH), 148.0, 150.2, 150.8 (CH), 154.3, 157.3, 161.2, 171.0; HRMS-ESI [(M + H)^+^] *m*/*z* calculated for C_24_H_24_N_4_O_4_: 433.1870, found: 433.1869.

6,7-dimethoxy-4-(4-(4-(trifluoromethyl)benzoyl)piperazin-1-yl)quinoline-3-carbonitrile (**5d**)

The yield was 83% (129 mg); Mp 147–148 °C; ^1^HNMR (CDCl_3_) δ: 3.53–4.01 (m, 8H, 4 × CH_2_), 4.03 (s, 3H, OCH_3_), 4.06 (s, 3H, OCH_3_), 7.26 (s, 1H, H-5), 7.41 (s, 1H, H-8), 7.61 (d, 2H, J = 7.9 Hz, H-2′, H-6′), 7.75 (d, 2H, J = 7.9 Hz, H-3′, H-5′), 8.67 (s, 1H, H-2); ^13^CNMR (CDCl_3_) δ: 51.8 (CH_2_), 56.1 (CH_3_), 56.4 (CH_3_), 96.8, 101.6 (CH), 109.1 (CH), 118.3, 118.4, 123.6 (q, J_C–F_ = 272.5 Hz, C), 125.8 (q, J_C–F_ = 4.0 Hz, CH), 127.6 (CH), 132.0 (q, J_C–F_ = 33.1 Hz), 138.6, 148.0, 150.3, 150.7 (CH), 154.3, 157.1, 169.4; HRMS-ESI [(M + H)^+^] *m*/*z* calculated for C_24_H_21_F_3_N_4_O_3_: 471.1639, found: 471.1640.

4-(4-(4-chlorobenzoyl)piperazin-1-yl)-6,7-dimethoxyquinoline-3-carbonitrile (**5e**)

The yield was 92% (133 mg); Mp 199–200 °C; ^1^HNMR (CDCl_3_) δ: 3.53–4.01 (m, 8H, 4 × CH_2_), 4.02 (s, 3H, OCH_3_), 4.05 (s, 3H, OCH_3_), 7.25 (s, 1H, H-5), 7.40 (s, 1H, H-8), 7.44 (s, 4H, H-2′, H-3′, H-5′, H-6′), 8.66 (s, 1H, H-2); ^13^CNMR (CDCl_3_) δ: 51.9 (CH_2_), 56.1 (CH_3_), 56.4 (CH_3_), 96.8, 101.8 (CH), 109.2 (CH), 118.4, 118.4, 128.8 (CH), 129.0 (CH), 133.4, 136.4, 148.1, 150.3, 150.8 (CH), 154.3, 157.2, 169.9; HRMS-ESI [(M + H)^+^] *m*/*z* calculated for C_23_H_21_ClN_4_O_3_: 437.1375, found: 437.1377.

4-(4-(4-fluorobenzoyl)piperazin-1-yl)-6,7-dimethoxyquinoline-3-carbonitrile (**5f**)

The yield was 70% (97 mg); Mp 208–209 °C; ^1^HNMR (CDCl_3_) δ: 3.65 (t, 4H, J = 4.9 Hz, 2 × CH_2_), 3.90 (br s, 4H, 2 × CH_2_), 4.03 (s, 3H, OCH_3_), 4.06 (s, 3H, OCH_3_), 7.11–7.21 (m, 2H, H-3′, H-5′), 7.26 (s, 1H, H-5), 7.41 (s, 1H, H-8), 7.46–7.56 (m, 2H, H-2′, H-6′), 8.67 (s, 1H, H-2); ^13^CNMR (CDCl_3_) δ: 51.9 (CH_2_), 56.0 (CH_3_), 56.4 (CH_3_), 96.6, 101.7 (CH), 109.1 (CH), 115.8 (d, J_C–F_ = 21.8 Hz, CH), 118.3, 118.4, 129.6 (d, J_C–F_ = 8.5 Hz, CH), 131.0 (d, J_C–F_ = 3.5 Hz, C), 148.0, 150.2, 150.7 (CH), 154.3, 157.2, 163.6 (d, J_C–F_ = 250.8 Hz, CH), 170.0; HRMS-ESI [(M + H)^+^] *m*/*z* calculated for C_23_H_21_FN_4_O_3_: 421.1670, found: 421.1671.

4-(4-(4-bromobenzoyl)piperazin-1-yl)-6,7-dimethoxyquinoline-3-carbonitrile (**5g**)

The yield was 90% (143 mg); Mp 203–204 °C; ^1^HNMR (CDCl_3_) δ: 3.64 (t, 4H, J = 5.1 Hz, 2 × CH_2_), 3.72–3.94 (br m, 4H, 2 × CH_2_), 4.02 (s, 3H, OCH_3_), 4.05 (s, 3H, OCH_3_), 7.25 (s, 1H, H-5), 7.37 (d, 2H, J = 8.5 Hz, H-3′, H-5′), 7.41 (s, 1H, H-8), 7.61 (d, 2H, J = 8.5 Hz, H-2′, H-6′), 8.66 (s, 1H, H-2); ^13^CNMR (CDCl_3_) δ: 51.9 (CH_2_), 56.1 (CH_3_), 56.4 (CH_3_), 96.8, 101.8 (CH), 109.2 (CH), 118.4, 118.4, 124.6, 129.0 (CH), 132.0 (CH), 133.9, 148.0, 150.3, 150.8 (CH), 154.3, 157.2, 169.9; HRMS-ESI [(M + H)^+^] *m*/*z* calculated for C_23_H_21_BrN_4_O_3_: 481.0870, found: 481.0872.

6,7-dimethoxy-4-(4-(3,4,5-trimethoxybenzoyl)piperazin-1-yl)quinoline-3-carbonitrile (**5h**)

The yield was 97% (158 mg); Mp 162–163 °C; ^1^HNMR (CDCl_3_) δ: 3.64 (t, J = 5.0 Hz, 4H, 2 × CH_2_), 3.85–3.94 (m, 13H, 2 × CH_2_, 3 × OCH_3_), 4.02 (s, 3H, OCH_3_), 4.05 (s, 3H, OCH_3_), 6.70 (s, 2H, H-2′, H-6′), 7.26 (s, 1H, H-5), 7.40 (s, 1H, H-8), 8.66 (s, 1H, H-2); ^13^CNMR (CDCl_3_) δ: 52.0 (CH_2_), 56.1 (CH_3_), 56.4 (CH_3_), 56.4 (CH_3_), 60.9 (CH_3_), 96.8, 101.8 (CH), 104.5 (CH), 109.1 (CH), 118.4, 118.4, 130.4, 139.6, 148.0, 150.3, 150.7 (CH), 153.5, 154.3, 157.3, 170.7; HRMS-ESI [(M + H)^+^] *m*/*z* calculated for C_26_H_28_N_4_O_6_: 493.2082, found: 493.2084.

4-(4-(3-chloro-4-fluorobenzoyl)piperazin-1-yl)-6,7-dimethoxyquinoline-3-carbonitrile (**5i**)

The yield was 94% (141 mg); Mp 204–205 °C; ^1^HNMR (CDCl_3_) δ: 3.65 (t, 4H, J = 4.9 Hz, 2 × CH_2_), 3.90 (br s, 4H, 2 × CH_2_), 4.03 (s, 3H, OCH_3_), 4.06 (s, 3H, OCH_3_), 7.22–7.27 (m, 2H, H-5, H-2′), 7.39 (ddd, 1H, J = 6.6, 4.5, 2.3 Hz, H-5′), 7.41 (s, 1H, H-8), 7.58 (dd, 1H, J = 6.9, 2.1 Hz, H-6′), 8.67 (s, 1H, H-2); ^13^CNMR (CDCl_3_) δ: 51.8 (CH_2_), 56.1 (CH_3_), 56.4 (CH_3_), 96.8, 101.7 (CH), 109.2 (CH), 117.0 (d, J_C–F_ = 21.9 Hz, CH), 118.4 (d, J_C–F_ = 2.3 Hz), 121.8, 121.9 (d, J_C–F_ = 16.8 Hz), 127.4 (d, J_C–F_ = 7.7 Hz, CH), 130.1 (CH), 132.2 (d, J_C–F_ = 3.8 Hz), 148.1, 150.3, 150.7 (CH), 154.4, 157.1, 159.1 (d, J_C–F_ = 254 Hz), 168.6; HRMS-ESI [(M + H)^+^] *m*/*z* calculated for C_23_H_20_ClFN_4_O_3_: 455.1281, found: 455.1281.

4-(4-(4-(dimethylamino)benzoyl)piperazin-1-yl)-6,7-dimethoxyquinoline-3-carbonitrile (**5j**)

The yield was 45% (66 mg); Mp 167–169 °C; ^1^HNMR (CDCl_3_) δ: 3.03 (s, 6H, 2 × CH_3_), 3.66 (t, 4H, J = 4.9 Hz, 2 × CH_2_), 3.93 (br s, 4H, 2 × CH_2_), 4.02 (s, 3H, OCH_3_), 4.05 (s, 3H, OCH_3_), 6.71 (d, 2H, J = 8.9 Hz, H-3′, H-5′), 7.27 (s, 1H, H-5), 7.40 (s, 1H, H-8), 7.44 (d, 2H, J = 8.9 Hz, H-2′, H-6′), 8.65 (s, 1H, H-2); ^13^CNMR (CDCl_3_) δ: 40.2 (CH_3_), 52.1 (CH_2_), 56.1 (CH_3_), 56.4 (CH_3_), 96.4, 102.1 (CH), 109.1 (CH), 111.2 (CH), 118.3, 118.6, 121.5, 129.6 (CH), 147.9, 150.1, 150.9 (CH), 151.8, 154.2, 157.5, 171.9; HRMS-ESI [(M + H)^+^] *m*/*z* calculated for C_25_H_27_N_5_O_3_: 446.2187, found: 446.2188.

4-(4-(2,3-dichlorobenzoyl)piperazin-1-yl)-6,7-dimethoxyquinoline-3-carbonitrile (**5k**)

The yield was 86% (134 mg); Mp 194–195 °C. ^1^HNMR (CDCl_3_) δ: 3.41–3.82 (m, 7H), 4.03 (s, 3H, OCH_3_), 4.05 (s, 3H, OCH_3_), 4.13–4.25 (m, 1H), 7.25 (s, 1H, H-5), 7.27 (dd, 1H, J = 7.7, 1.7 Hz, H-6′), 7.33 (t, 1H, J = 7.7 Hz, H-5′), 7.41 (s, 1H, H-8), 7.54 (dd, J = 7.9, 1.7 Hz, 1H, H-4′), 8.66 (s, 1H, H-2); ^13^CNMR (CDCl_3_) δ: 42.4 (CH_2_), 47.4 (CH_2_), 51.6 (CH_2_), 51.9 (CH_2_), 56.1 (CH_3_), 56.4 (CH_3_), 96.8, 101.7, 109.2 (CH), 118.3, 118.4, 126.0 (CH), 128.3 (CH), 128.9, 131.2 (CH), 133.9, 137.3, 148.1, 150.3, 150.7 (CH), 154.3, 157.1, 166.4; HRMS-ESI [(M + H)^+^] *m*/*z* calculated for C_23_H_20_Cl_2_N_4_O_3_: 471.0985, found: 471.0986.

### 3.2. Biology

#### 3.2.1. Evaluation of Bacteriostatic and Growth-Inhibitory Properties of Novel 6,7-Dimethoxy-4-piperazinyl Quinoline Derivatives

A bacterial survival assay assessed the antimicrobial activity of 6,7-dimethoxy-4-piperazinyl quinoline derivatives **5a**–**k** against the reference pathogens *S. aureus* ATCC 25923 and *P. aeruginosa* ATCC 10145. In summary, a single colony of the strain was selected and pre-cultured for approximately 16 h at 37 °C with shaking at 180 rpm in a Luria-Bertani (LB) medium, which consists of 10 g/L NaCl, 5 g/L yeast extract, and 10 g/L tryptone. When required, this medium was solidified with 15 g/L bacteriological agar. Bacterial cells were then inoculated at a dilution of 10^−5^ into the LB medium supplemented with increasing concentrations of the 4-piperazinylquinoline derivatives **5a**–**k**, ranging from 0 to 50 μM, and incubated under the same conditions for 24 h. The number of viable bacteria (colony-forming units per milliliter, CFU/mL) that survived the treatment was determined using the spot plate count method and compared to untreated controls.

The same procedure was applied to assess the efficacy of the quinoline compounds after 0, 3, and 6 h of treatment at 2.5, 15, and 50 μM. Data are shown as the average (n = 3) CFU/mL on a logarithmic scale (Log_10_) with standard deviation (SD).

#### 3.2.2. Minimum Inhibitory Concentration (MIC) Determination

To determine the MIC of the 6,7-dimethoxy-4-piperazinyl quinoline derivatives **5a**–**k** against *S. aureus* ATCC 25923, a 10^−5^ dilution of overnight bacterial culture in LB medium was incubated with increasing concentrations (0.5, 5, 10, and 50 μM) of the 6,7-dimethoxy-4-piperazinyl quinoline derivatives **5k**. The optical density of the cultures at 600 nm, an indirect measure of bacterial growth, was recorded at 0 and after 3 and 6 h of exposure using a spectrophotometer Jasco 7850 (Lecco, Italy, Jasco Europe). Optical density values were corrected by subtracting the optical density of the LB medium containing the same concentration of the novel 6,7-dimethoxy-4-piperazinyl quinoline derivatives. The experiment was performed in triplicate to ensure the consistency and accuracy of the results.

#### 3.2.3. Cell Viability Assay

*S. aureus* ATCC 25923 cells were cultured for 0, 3, and 6 h either in the absence or presence of 2.5, 15, and 50 μM of the 6,7-dimethoxy-4-piperazinylquinoline derivatives. Following incubation, the cells were harvested and rinsed three times with PBS before being stained with the Live/Dead^®^ BacLight™ kit (Molecular Probes, Merck Life Science S.r.l., Milan, Italy) for 30 min at room temperature following the protocol of the producer. This kit utilizes two fluorescent dyes: SYTO 9, which emits green fluorescence and stains all live bacterial cells, and propidium iodide, which emits red fluorescence and selectively stains dead cells with compromised membranes. Each stained bacterial sample was visualized using a Leica DM2500 fluorescence microscope (Wetzlar, Germany) with a 63× objective. Fluorescence microscopy images were taken with red and green fluorescence filters and merged using ImageJ software 1.53a (Bethesda, MD, USA).

### 3.3. In Silico Studies

#### 3.3.1. ADMET, Drug-Likeness, and Medicinal Chemistry Friendliness Parameters Prediction

The QikProp tool, available in the Maestro suite-Schrödinger (Schrödinger Release 2017-1: QikProp, Schrödinger, LLC, New York, NY, 2017), was used to predict the ADME properties (absorption, distribution, metabolism, and excretion) of the input structures [30]. It calculates significant physical and chemical descriptors regarding size, polarity, lipophilia, structural features, and pharmaceutical properties. Input structures were initially prepared and optimized as .sdf files using the LigPrep task (as mentioned in Section 3.3.1) before being processed in standard mode with QikProp.

Selected ADME parameters, along with bioavailability radar and BOILED-Egg plots, were instead generated via the SwissADME server (http://www.swissadme.ch, accessed on 20 December 2024) by submitting the SMILES strings for each compound, as detailed in [29,31].

#### 3.3.2. Ligand Preparation

LigPrep, a tool part of the Maestro Suite from Schrödinger (Schrödinger Release 2017-2, LigPrep; Schrödinger, LLC: New York, NY, USA, 2017), was employed for preparing small-molecule ligand structures for structure-based virtual screening. Both **5k** and reference target inhibitors were imported as .sdf files and processed following the protocol as follows. It generated corresponding low-energy 3D conformations of the molecule by exploring tautomeric and ionization states as well as ring conformations and stereoisomers based on the provided input. For each ligand, all possible tautomers and stereoisomers were produced while preserving specified chiralities. Unwanted species, such as water or counter ions, were eliminated from the dataset. Ionization states were calculated over a pH range of 7.0 ± 0.4 using the default Epik method, while the specified ionization states in the input structure were maintained. Following this, the energy of the ligands was minimized using the Optimized Potentials for Liquid Simulations (OPLS 2005) force field [53,54]. The output structures were then readily available for virtual screening.

#### 3.3.3. Protein Retrieval and Preparation

The X-ray crystal structures of the targets studied were obtained from the Protein Data Bank in .pdb format [55,56], selecting S. aureus as the “organism” and corresponding to the following PDB codes: tyrosyl-tRNA synthetase (TyrRS, PDB id 1JIJ [48]); FtsZ (PDB id 4DXD [51]); Pyruvate Kinase (PDB id 3T0T [49]); DNA gyrase B (PDB id 3G7B [45]); C30 carotenoid dehydrosqualene synthase (CrtM, PDB id 2ZCQ [50]); Thymidylate Kinase (TMK, PDB id [46]); dehydrofolate reductase (DHFR, PDB id 3FYW [47]).

The standard .pdb file format is not ideal for direct application in molecular modeling studies due to the inclusion of water, solvents, buffer molecules, metal ions, and cofactors, co-resolved during the crystallographic experiments. To address this issue, the proteins were prepared and refined using the Protein Preparation Wizard Task within the Schrödinger software utilizing the default settings (Schrödinger Suite 2017-2 Protein Preparation Wizard; Epik, Schrödinger, LLC: New York, NY, USA, 2017) [54,57,58]. This process comprised several sequential steps. Initially, each pdb structure was imported into Maestro and underwent pre-processing as follows (Preprocess Panel): bond orders, including those for the Het group, were assigned; all water molecules were removed; the protonation states of heteroatoms were determined using the Epik tool (with pH set to biologically relevant values of 7.0 ± 0.4); disulfide bonds were formed; and missing loops and side chains adjacent to the binding site were modeled using Prime. In the following step (Analyze Panel), redundant subunits in multimeric complexes were eliminated to streamline the structures. Finally, in the last step (Optimize and Minimize Panel), the hydrogen bond network was optimized, and the structures underwent a restrained energy minimization step (with a root mean square deviation (RMSD) of 0.3 Å set as the termination criterion) utilizing the OPLS 2005 force field [54].

#### 3.3.4. Structure-Based Studies: Induced Fit Docking (IFD) Simulations

An Induced Fit Docking (IFD) simulation was conducted utilizing Schrödinger’s Induced Fit Docking tool, known for its accuracy in considering the flexibility of both the ligand and receptor. The validated IFD protocol from Schrödinger was implemented on the receptor model, which had been previously refined with the Protein Preparation Wizard. The IFD score was determined using the formula IFD score = 1.0 × Glide Gscore + 0.05 × Prime Energy. This score integrates the protein–ligand interaction energy with the overall system energy and was employed to rank the IFD poses under default settings [59,60,61,62,63]. For each target, the co-crystallized reference ligand and other significative reference inhibitors were docked for comparison. The docking procedure effectively re-docked the original ligands into the receptor binding sites, achieving a root mean square deviation (RMSD) of under 0.50 Å.

#### 3.3.5. Structure-Based Studies: Molecular Dynamics Simulation

Molecular dynamics simulations were conducted for the best complexes of IFD studies to explore how the solvent environment impacts the stability of the protein–ligand complex structure. These simulations utilized the explicit-solvent molecular dynamics capability of the Desmond package (Schrödinger Release 2023-4: Desmond Molecular Dynamics System, D. E. Shaw Research, New York, NY, 2024. Maestro-Desmond Interoperability Tools, Schrödinger, New York, NY, 2023), integrated into the Maestro version 13.8.155, MMshare Version 6.4.195, Release 2023-4 [64], available for Platform Linux-x8564. Simulations were run on a Dell Precision 7960 Tower with an Intel^®^ Xeon^®^ w9-3475X processor (Santa Clara, CA, USA) and NVIDIA GPU (Santa Clara, CA, USA), all operating on Ubuntu 22.04.4 LTS 64-bit. Each thermodynamic system occupied a defined three-dimensional space, with atoms represented as particles. Starting positions were taken from the best-docked poses of the **5k** in complex with the selected receptor, as identified in previous sections. The System Builder panel was used to prepare each complex, employing the simple point charge (SPC) water model for solvation and neutralization. The simulation box was automatically sized using an orthorhombic shape with a 10 Å buffer distance from the solute. The simulations ran for 100 ns with the default OPLS4 force field, yielding approximately 1000 frames. Standard thermodynamic parameters were applied in a constant-temperature-constant-pressure (NPT) ensemble at 300 K and 1013.25 bar. Prior to simulation, systems were minimized and equilibrated. Trajectories were visually inspected with the Trajectory Player, and reliability was confirmed through the Simulation Quality Analysis panel by monitoring total and potential energy, temperature, pressure, and volume stability. Graphical outputs were generated to illustrate geometric properties, including root mean square deviation (RMSD) and root mean square fluctuation (RMSF) for both proteins and ligands.

## 4. Conclusions

In this study, a new series of 6,7-dimethoxy-4-piperazinylquinoline-3-carbonitrile derivatives (**5a**–**k**) was designed using a molecular hybridization approach that combines three pharmacophoric moieties—quinoline, piperazine, and the hydrophobic benzoyl portion—known for their roles as antimicrobial agents.

The designed compound library was preliminarily evaluated in silico to predict key physicochemical, ADME, and drug-likeness properties, demonstrating an optimal hydrophilicity–lipophilicity balance, high membrane permeability, complete predicted oral absorption, and no violations of commonly used drug-likeness rules and filters.

Based on these promising results, a multistep synthetic strategy was developed, involving initial construction of the quinoline core, sequential addition of the piperazine fragment, and final introduction of variously substituted benzoyl groups. The resulting compounds were obtained in high yields and adequate purity for subsequent in vitro testing.

The complete set of compounds (**5a**–**k**) was tested against two representative bacterial strains, *Staphylococcus aureus* ATCC 25923 (Gram-positive) and *Pseudomonas aeruginosa* ATCC 10145 (Gram-negative). Notably, compound **5k** exhibited promising antibiotic activity against *S. aureus*, with an MIC in the micromolar range comparable to previously reported antimicrobial piperazinyl-quinoline compounds (compounds **1**–**4**, see Introduction). A dose- and time-dependent effect was also observed at concentrations above 15 μM. Fluorescence imaging revealed morphological changes in *S. aureus* cells treated with **5k**, indicating that at 15 μM, bacterial cell membrane integrity began to deteriorate. We hypothesize that the enhanced lipophilicity and membrane permeability predicted in silico provided by the two chlorine atoms facilitated higher membrane penetration, potentially leading to disruption of physiological functions, inhibition of replication-essential enzymes, and ultimately cell death [65,66,67,68,69].

On the other hand, none of the compounds, including **5k**, showed activity against the representative Gram-negative strain *P. aeruginosa* ATCC 10145. The differing membrane composition between Gram-positive and Gram-negative bacteria, with an additional outer membrane layer in the latter, may account for the limited penetration of this compound into Gram-negative cells. In this context, the so-called “entry rules” for a small molecule intended to accumulate in Gram-negative bacteria could be considered for the lead optimization process. These include reduction of logP and globularity as well as the introduction of primary charged amino side groups [70,71].

Finally, in silico structure-based docking and molecular dynamics studies were conducted on selected key targets from *S. aureus*, identifying potential strong interactions with tyrosyl-tRNA synthetase, DNA gyrase B, and pyruvate kinase.

Overall, the molecular hybridization approach used in this study yielded compound **5k** as the most promising antibiotic derivative of the series. Both the in vitro and in silico investigations provide a foundation for a lead optimization strategy and the development of new, more active, and broader-spectrum derivatives.

## Figures and Tables

**Table 1:** Key predicted ADME properties calculated through SwissADME and QikProp tools for compounds **5a**–**k**.

Compound *	MW	C LogP_o/w_	TPSA	Csp^3^	RB	ESOL LogS	QPPCaco
**5a**	402.45	2.56	78.69	0.26	5	−4.23	860.5
**5b**	416.47	2.92	78.69	0.29	5	−4.53	860.3
**5c**	432.47	2.54	87.92	0.29	6	−4.30	858.4
**5d**	470.44	3.60	78.69	0.29	6	−5.10	917.9
**5e**	436.89	3.10	78.69	0.26	5	−4.82	859.3
**5f**	420.44	2.84	78.69	0.26	5	−4.39	859.2
**5g**	481.34	3.18	78.69	0.26	5	−5.14	789.5
**5h**	492.52	2.55	106.38	0.35	8	−4.47	938.4
**5i**	454.88	3.32	78.69	0.26	5	−4.99	710.91
**5j**	445.51	2.57	81.93	0.32	6	−4.47	828.9
**5k**	471.34	3.55	78.69	0.26	5	−5.42	1013.8

* MW, molecular weight; C logP_o/w_, consensus log P octanol/water (average value from five different models, iLogP, XLogP3, WLogP, MLogP, SILICOS-IT); TPSA, total polar surface area in Å; Csp^3^, fraction of carbons with sp^3^ hybridization; RB, rotatable bonds; LogS, solubility scale; QPPCaco, predicted apparent Caco-2 cell permeability in nm/s.

**Table 2:** Drug-likeness rules and filters for 4-piperazinylquinolines **5a**–**k**.

Compound	QP Stars *	LRoF	GV	VV	EV	MV	PAINS	Brenk Alerts
**5a**	0	0	0	0	0	0	0	0
**5b**	0	0	0	0	0	0	0	0
**5c**	0	0	0	0	0	0	0	0
**5d**	1	0	0	0	0	0	0	0
**5e**	0	0	0	0	0	0	0	0
**5f**	0	0	0	0	0	0	0	0
**5g**	1	0	1	0	0	0	0	0
**5h**	0	0	2	0	0	0	0	0
**5i**	0	0	0	0	0	0	0	0
**5j**	0	0	1	0	0	0	0	0
**5k**	1	0	1	0	0	0	0	0

* QP stars: QikProp stars (number of property or descriptor values that fall outside the 95% range of similar values computed for known drugs); LRoF: Lipinski Rule of Five; GV: Ghose Violations; VV: Veber Violations; EV: Egan Violations; MV: Muegge Violations; PAINS: Pan-Assay Interference Compounds.

**Table 3:** Induced Fit Docking results for compound **5k** against the seven selected *S. aureus* targets (PDB codes listed), showing IFD, glide, and prime energy scores. The corresponding results referring to the reference inhibitors are presented for each target as comparative ranges.

Target	PDB	Compound	IFD Score	Glide Score	Prime Energy
**Tyrosyl**–tRNA synthetase (TyrRS) *	1JIJ [48]	Ref. inhibitors ^#^	−676.10 ÷ −681.22	−9.552 ÷ −11.473	−13,327.4 ÷ −13,390.2
		**5k**	−678.81	−9.268	−13,390.3
**Pyruvate** Kinase (PK) *	3T0T [49]	Ref. inhibitors	−2473.28 ÷ −2476.14	−10.038 ÷ −11.023	−49,244.4 ÷ −49,320.6
		**5k**	−2473.46	−9.572	−49,277.2
Gyrase B *	3G7B [45]	Ref. inhibitors	−442.53 ÷ −445.73	−6.890 ÷ −7.911	−8704.1 ÷ −8755.1
		**5k**	−442.92	−8.751	−8682.8
**FtsZ**	4DXD [51]	Ref. inhibitors	−696.44 ÷ −696.62	−11.082 ÷ −11.814	−13,696.0 ÷ −13,707.3
		**5k**	−693.60	−9.507	13,681.3
**CrtM**	2ZCQ [50]	Ref. inhibitors	−686.06 ÷ −688.91	−10.927 ÷ −13.583	−13,429.6 ÷ −13,506.6
		**5k**	−682.43	−10.927	−13,429.6
**Thymidylate Kinase (TMK)**	4HLC [46]	Ref. inhibitors	−453.73 ÷ −455.97	−8.513 ÷ −10.406	−8823.6 ÷ −8910.8
		**5k**	−449.39	−8.185	
**Dihydrofolate reductase (DHFR)**	3FYW [47]	Ref. inhibitors	−373.07 ÷ −375.62	−8.021 ÷ −9.797	−7291.2 ÷ −7306.7
		**5k**	−364.46	−7.644	−7135.7

* Target proteins for which **5k** has reported IFD, glide, and prime energy score values comparable to/higher than that of the reference inhibitors and selected for molecular dynamic simulations. ^#^ List of reference inhibitors: CID446499, CID446497, CID446500, CID446498 (tyrosyl–tRNA synthetase); CID10624851, CID135642239 (pyruvate kinase); CID11718729, CID44608008, CID2744426, CID54759160, CID153541355 (DNA gyrase B); CID90135655, CID25016417 (Ftsz); CID56928041, CID44182294, CID24748047, CID56928042 (CrtM); CID66545765, CID66553127, CID66553128 (thymidylate kinase); CID10247560, CID53346505, CID42627761, CID5578 (DHFR).

## Data Availability

Data are contained within the article or Supplementary Material.

## Supplementary Materials

The following supporting information can be downloaded at https://www.mdpi.com/article/10.3390/molecules30010028/s1, S1, ADMET and drug-likeness parameters predictions, SwissADME and QikProp output files (.xlsx); S2–S4, Induced Fit Docking (IFD) and molecular dynamic simulations additional data, Supporting material for in vitro assays on *S. aureus*, ^1^H and ^13^CNMR spectra for compounds **10** and **5a**–**k** (.pdf).

## References

[1] Aatif M., Raza M.A., Javed K., Nashre-Ul-Islam S.M., Farhan M., Alam M.W. (2022). Potential Nitrogen-Based Heterocyclic Compounds for Treating Infectious Diseases: A Literature Review. Antibiotics.

[2] Heravi M.M., Zadsirjan V. (2020). Prescribed drugs containing nitrogen heterocycles: An overview. RSC Adv..

[3] Dube P.S., Legoabe L.J., Beteck R.M. (2023). Quinolone: A versatile therapeutic compound class. Mol. Divers..

[4] Senerovic L., Opsenica D., Moric I., Aleksic I., Spasić M., Vasiljevic B. (2020). Quinolines and Quinolones as Antibacterial, Antifungal, Anti-virulence, Antiviral and Anti-parasitic Agents. Adv. Exp. Med. Biol..

[5] Kumar P. (2020). A review on quinoline derivatives as anti-methicillin resistant *Staphylococcus aureus* (MRSA) agents. BMC Chem..

[6] Tahir S., Mahmood T., Dastgir F., Haq I.U., Waseem A., Rashid U. (2019). Design, synthesis and anti-bacterial studies of piperazine derivatives against drug resistant bacteria. Eur. J. Med. Chem..

[7] Faizan M., Kumar R., Mazumder A., Salahuddin, Kukreti N., Kumar A., Chaitanya M.V.N.L. (2024). The medicinal chemistry of piperazines: A review. Chem. Biol. Drug Des..

[8] Patil M., Noonikara Poyil A., Joshi S.D., Patil S.A., Bugarin A. (2019). Design, synthesis, and molecular docking study of new piperazine derivative as potential antimicrobial agents. Bioorg. Chem..

[9] Bérubé G. (2016). An overview of molecular hybrids in drug discovery. Expert. Opin. Drug Discov..

[10] Ivasiv V., Albertini C., Gonçalves A.E., Rossi M., Bolognesi M.L. (2019). Molecular Hybridization as a Tool for Designing Multitarget Drug Candidates for Complex Diseases. Curr. Top. Med. Chem..

[11] Elebiju O.F., Ajani O.O., Oduselu G.O., Ogunnupebi T.A., Adebiyi E. (2022). Recent advances in functionalized quinoline scaffolds and hybrids-Exceptional pharmacophore in therapeutic medicine. Front. Chem..

[12] Patel K.B., Kumari P. (2022). A review: Structure-activity relationship and antibacterial activities of Quinoline based hybrids. J. Mol. Struct..

[13] Bala I.A., Al Sharif O.F., Asiri A.M., El-Shishtawy R.M. (2024). Quinoline: A versatile bioactive scaffold and its molecular hybridization. Results Chem..

[14] Insuasty D., Vidal O., Bernal A., Marquez E., Guzman J., Insuasty B., Quiroga J., Svetaz L., Zacchino S., Puerto G. (2019). Antimicrobial Activity of Quinoline-Based Hydroxyimidazolium Hybrids. Antibiotics.

[15] Verma S., Lal S., Narang R., Sudhakar K. (2023). Quinoline Hydrazide/Hydrazone Derivatives: Recent Insights on Antibacterial Activity and Mechanism of Action. ChemMedChem.

[16] El-Azzouny A.M.A.E., Aboul-Enein M.N., Hamissa M.F. (2020). Structural and biological survey of 7-chloro-4-(piperazin-1-yl)quinoline and its derivatives. Drug Dev. Res..

[17] Banu S., Bollu R., Naseema M., Gomedhika P.M., Nagarapu L., Sirisha K., Kumar C.G., Gundasw S.K. (2018). A novel templates of piperazinyl-1,2-dihydroquinoline-3-carboxylates: Synthesis, anti-microbial evaluation and molecular docking studies. Bioorg. Med. Chem. Lett..

[18] Pathak P., Thakur A., Bhat H.R., Singh U.P. (2015). Hybrid 4-Aminoquinoline-1,3,5-triazine Derivatives: Design, Synthesis, Characterization, and Antibacterial Evaluation. J. Heterocycl. Chem..

[19] Subramanian S., Eswaran S. (2020). Design, synthesis and study of antibacterial and antitubercular activity of quinoline hydrazone hybrids. Heterocycl. Comm..

[20] Gnanavelu K., Vinay Kumar K.S., Eswaran S., Sivashanmugam K. (2023). Novel quinoline-piperazine hybrids: The design, synthesis and evaluation of antibacterial and antituberculosis properties. RSC Med. Chem..

[21] Lauria A., La Monica G., Bono A., Martorana A. (2021). Quinoline anticancer agents active on DNA and DNA-interacting proteins: From classical to emerging therapeutic targets. Eur. J. Med. Chem..

[22] Martorana A., La Monica G., Lauria A. (2020). Quinoline-Based Molecules Targeting c-Met, EGF, and VEGF Receptors and the Proteins Involved in Related Carcinogenic Pathways. Molecules.

[23] Mingoia F., Di Sano C., D’Anna C., Fazzari M., Minafra L., Bono A., La Monica G., Martorana A., Almerico A.M., Lauria A. (2023). Synthesis of new antiproliferative 1,3,4-substituted-pyrrolo [3,2-c]quinoline derivatives, biological and in silico insights. Eur. J. Med. Chem..

[24] La Monica G., Pizzolanti G., Baiamonte C., Bono A., Alamia F., Mingoia F., Lauria A., Martorana A. (2023). Design and Synthesis of Novel Thieno[3,2-*c*]quinoline Compounds with Antiproliferative Activity on RET-Dependent Medullary Thyroid Cancer Cells. ACS Omega.

[25] Dulsat J., López-Nieto B., Estrada-Tejedor R., Borrell J.I. (2023). Evaluation of Free Online ADMET Tools for Academic or Small Biotech Environments. Molecules.

[26] Wang J., Urban L., Bojanic D. (2007). Maximising use of in vitro ADMET tools to predict in vivo bioavailability and safety. Expert. Opin. Drug Metab. Toxicol..

[27] Vrbanac J., Slauter R. (2016). ADME in Drug Discovery. A Comprehensive Guide to Toxicology in Nonclinical Drug Development.

[28] Stegemann S., Leveiller F., Franchi D., de Jong H., Lindén H. (2007). When poor solubility becomes an issue: From early stage to proof of concept. Eur. J. Pharm. Sci..

[29] Daina A., Michielin O., Zoete V. (2017). SwissADME: A free web tool to evaluate pharmacokinetics, drug-likeness and medicinal chemistry friendliness of small molecules. Sci. Rep..

[30] (2017). Schrödinger Release 2017-1: QikProp.

[31] Daina A., Zoete V. (2016). A BOILED-Egg To Predict Gastrointestinal Absorption and Brain Penetration of Small Molecules. ChemMedChem.

[32] Lipinski C.A., Lombardo F., Dominy B.W., Feeney P.J. (2001). Experimental and computational approaches to estimate solubility and permeability in drug discovery and development settings. Adv. Drug Deliv. Rev..

[33] Ghose A.K., Viswanadhan V.N., Wendoloski J.J. (1999). A knowledge-based approach in designing combinatorial or medicinal chemistry libraries for drug discovery. 1. A qualitative and quantitative characterization of known drug databases. J. Comb. Chem..

[34] Veber D.F., Johnson S.R., Cheng H.Y., Smith B.R., Ward K.W., Kopple K.D. (2002). Molecular properties that influence the oral bioavailability of drug candidates. J. Med. Chem..

[35] Egan W.J., Merz K.M., Baldwin J.J. (2000). Prediction of drug absorption using multivariate statistics. J. Med. Chem..

[36] Muegge I., Heald S.L., Brittelli D. (2001). Simple selection criteria for drug-like chemical matter. J. Med. Chem..

[37] Baell J.B., Holloway G.A. (2010). New substructure filters for removal of pan assay interference compounds (PAINS) from screening libraries and for their exclusion in bioassays. J. Med. Chem..

[38] Brenk R., Schipani A., James D., Krasowski A., Gilbert I.H., Frearson J., Wyatt P.G. (2008). Lessons learnt from assembling screening libraries for drug discovery for neglected diseases. ChemMedChem.

[39] Weyesa A., Mulugeta E. (2020). Recent advances in the synthesis of biologically and pharmaceutically active quinoline and its analogues: A review. RSC Adv..

[40] Wissner A., Berger D.M., Boschelli D.H., Floyd M.B., Greenberger L.M., Gruber B.C., Johnson B.D., Mamuya N., Nilakantan R., Reich M.F. (2000). 4-Anilino-6,7-dialkoxyquinoline-3-carbonitrile inhibitors of epidermal growth factor receptor kinase and their bioisosteric relationship to the 4-anilino-6,7-dialkoxyquinazoline inhibitors. J. Med. Chem..

[41] Capri F.C., Di Leto Y., Presentato A., Mancuso I., Scatassa M.L., Alduina R. (2024). Characterization of *Staphylococcus* Species Isolates from Sheep Milk with Subclinical Mastitis: Antibiotic Resistance, Enterotoxins, and Biofilm Production. Foodborne Pathog. Dis..

[42] Secretary E. (1970). OPINION 36: Designation of Strain ATCC 10145 as the Neotype Strain of Pseudomonas aeruginosa (Schroeter) Migula. Int. J. Syst. Bacteriol..

[43] Rubino S., Pibiri I., Minacori C., Alduina R., Di Stefano V., Orecchio S., Buscemi S., Girasolo M.A., Tesoriere L., Attanzio A. (2018). Synthesis, structural characterization, anti-proliferative and antimicrobial activity of binuclear and mononuclear Pt(II) complexes with perfluoroalkyl-heterocyclic ligands. Inorganica Chim. Acta.

[44] Saladino M.L., Markowska M., Carmone C., Cancemi P., Alduina R., Presentato A., Scaffaro R., Biały D., Hasiak M., Hreniak D. (2020). Graphene Oxide Carboxymethylcellulose Nanocomposite for Dressing Materials. Materials.

[45] Ronkin S.M., Badia M., Bellon S., Grillot A.L., Gross C.H., Grossman T.H., Mani N., Parsons J.D., Stamos D., Trudeau M. (2010). Discovery of pyrazolthiazoles as novel and potent inhibitors of bacterial gyrase. Bioorg. Med. Chem. Lett..

[46] Martínez-Botella G., Loch J.T., Green O.M., Kawatkar S.P., Olivier N.B., Boriack-Sjodin P.A., Keating T.A. (2013). Sulfonylpiperidines as novel, antibacterial inhibitors of Gram-positive thymidylate kinase (TMK). Bioorg. Med. Chem. Lett..

[47] Oefner C., Parisi S., Schulz H., Lociuro S., Dale G.E. (2009). Inhibitory properties and X-ray crystallographic study of the binding of AR-101, AR-102 and iclaprim in ternary complexes with NADPH and dihydrofolate reductase from *Staphylococcus aureus*. Acta Crystallogr. D Biol. Crystallogr..

[48] Qiu X., Janson C.A., Smith W.W., Green S.M., McDevitt P., Johanson K., Carter P., Hibbs M., Lewis C., Chalker A. (2001). Crystal structure of *Staphylococcus aureus* tyrosyl-tRNA synthetase in complex with a class of potent and specific inhibitors. Protein Sci..

[49] Axerio-Cilies P., See R.H., Zoraghi R., Worral L., Lian T., Stoynov N., Jiang J., Kaur S., Jackson L., Gong H. (2012). Cheminformatics-driven discovery of selective, nanomolar inhibitors for staphylococcal pyruvate kinase. ACS Chem. Biol..

[50] Liu C.I., Liu G.Y., Song Y., Yin F., Hensler M.E., Jeng W.Y., Nizet V., Wang A.H., Oldfield E. (2008). A cholesterol biosynthesis inhibitor blocks *Staphylococcus aureus* virulence. Science.

[51] Tan C.M., Therien A.G., Lu J., Lee S.H., Caron A., Gill C.J., Lebeau-Jacob C., Benton-Perdomo L., Monteiro J.M., Pereira P.M. (2012). Restoring methicillin-resistant *Staphylococcus aureus* susceptibility to β-lactam antibiotics. Sci. Transl. Med..

[52] Vincetti P., Caporuscio F., Kaptein S., Gioiello A., Mancino V., Suzuki Y., Yamamoto N., Crespan E., Lossani A., Maga G. (2015). Discovery of Multitarget Antivirals Acting on Both the Dengue Virus NS5-NS3 Interaction and the Host Src/Fyn Kinases. J. Med. Chem..

[53] (2017). Schrödinger Release 2017–2: LigPrep.

[54] Banks J.L., Beard H.S., Cao Y., Cho A.E., Damm W., Farid R., Felts A.K., Halgren T.A., Mainz D.T., Maple J.R. (2005). Integrated Modeling Program, Applied Chemical Theory (IMPACT). J. Comput. Chem..

[55] Berman H.M., Westbrook J., Feng Z., Gilliland G., Bhat T.N., Weissig H., Shindyalov I.N., Bourne P.E. (2000). The Protein Data Bank. Nucleic Acids Res..

[56] RCSB PDB. www.rcsb.org.

[57] (2017). Schrödinger Release 2017-2: Protein Preparation Wizard.

[58] Sastry G.M., Adzhigirey M., Day T., Annabhimoju R., Sherman W. (2013). Protein and ligand preparation: Parameters, protocols, and influence on virtual screening enrichments. J. Comput. Aided Mol. Des..

[59] Luo H.-J., Wang J.-Z., Deng W.-Q., Zou K. (2013). Induced-fit docking and binding free energy calculation on furostanol saponins from Tupistra chinensis as epidermal growth factor receptor inhibitors. Med. Chem. Res..

[60] Zhong H., Tran L.M., Stang J.L. (2009). Induced-fit docking studies of the active and inactive states of protein tyrosine kinases. J. Mol. Graph. Model..

[61] Wang H., Aslanian R., Madison V.S. (2008). Induced-fit docking of mometasone furoate and further evidence for glucocorticoid receptor 17alpha pocket flexibility. J. Mol. Graph. Model..

[62] Sherman W., Beard H.S., Farid R. (2006). Use of an induced fit receptor structure in virtual screening. Chem. Biol. Drug Des..

[63] Sherman W., Day T., Jacobson M.P., Friesner R.A., Farid R. (2006). Novel procedure for modeling ligand/receptor induced fit effects. J. Med. Chem..

[64] (2023). Schrödinger Release 2023-4: Desmond Molecular Dynamics System.

[65] Hooper D.C. (2001). Mechanisms of action of antimicrobials: Focus on fluoroquinolones. Clin. Infect. Dis..

[66] Murínová S., Dercová K. (2014). Response mechanisms of bacterial degraders to environmental contaminants on the level of cell walls and cytoplasmic membrane. Int. J. Microbiol..

[67] Sikkema J., de Bont J.A., Poolman B. (1995). Mechanisms of membrane toxicity of hydrocarbons. Microbiol. Rev..

[68] Treangen T.J., Maybank R.A., Enke S., Friss M.B., Diviak L.F., Karaolis D.K., Koren S., Ondov B., Phillippy A.M., Bergman N.H. (2014). Complete Genome Sequence of the Quality Control Strain *Staphylococcus aureus* subsp. aureus ATCC 25923. Genome Announc..

[69] Walton I.M., Cox J.M., Benson C.A., Patel D.G., Chen Y.S., Benedict J.B. (2016). The role of atropisomers on the photo-reactivity and fatigue of diarylethene-based metal-organic frameworks. New J. Chem..

[70] Richter M.F., Hergenrother P.J. (2019). The challenge of converting Gram-positive-only compounds into broad-spectrum antibiotics. Ann. N. Y. Acad. Sci..

[71] Saxena D., Maitra R., Bormon R., Czekanska M., Meiers J., Titz A., Verma S., Chopra S. (2023). Tackling the outer membrane: Facilitating compound entry into Gram-negative bacterial pathogens. Npj Antimicrob. Resist..

