# Supplementary material for: Novel Antibacterial 4-Piperazinylquinoline Hybrid Derivatives Against Staphylococcus aureus: Design, Synthesis, and In Vitro and In Silico Insights
Source: Molecules. 2024 Dec 25;30(1):28. doi: 10.3390/molecules30010028 (PMC11720749; doi:10.3390/molecules30010028)

Supporting Information

Novel Antibacterial 4-Piperazinylquinoline Hybrid Derivatives Against *Staphylococcus aureus*: Design, Synthesis, and In Vitro and In Silico Insights

Gabriele La Monica [1], Annamaria Gallo [1], Alessia Bono [1], Federica Alamia [1], Antonino Lauria [1,2], Rosa Alduina [1,2] and Annamaria Martorana [1,*]

1 Department of Biological, Chemical and Pharmaceutical Sciences and Technologies (STEBICEF), University of Palermo, Viale delle Scienze, 90128 Palermo, Italy; gabriele.lamonica01@unipa.it (G.L.M.); annamaria.gallo01@unipa.it (A.G.); alessia.bono01@unipa.it (A.B.); federica.alamia01@unipa.it (F.A.); antonino.lauria@unipa.it (A.L.); valeria.alduina@unipa.it (R.A.)

2 NBFC, National Biodiversity Future Center, Piazza Marina 61, 90133 Palermo, Italy

* Correspondence: annamaria.martorana@unipa.it

Contents

Sr. No.	Description	Page no.
S1.	ADMET and drug-likeness parameters predictions (SwissADME and QikProp output files)	**.xlsx file**
S2.	Induced Fit Docking (IFD) and molecular dynamic simulations additional data	**S2-S4**
S3.	Supporting material for in vitro assays on S. aureus	**S5-S6**
S4.	^{1}H and ^{13}C NMR spectra for compounds **10** and **5a-k**	**S7-S18**

S2. Induced Fit Docking molecular dynamic simulations additional data

Supporting Table S1. Full Induced Fit Docking results, including IFD, Glide and Prime Energy scores obtained for compound **5k** against the 7 investigated targets crucial for *Staphylococcus aureus* life cycle. For each target, the results related to the corresponding co-crystallized inhibitor as well as to other reference inhibitors are reported as controls. In red the 3 best performing targets considered for MD studies are highlighted.

Target	Compound	IFD Score	Glide score	Prime Energy
**Tyrosyl-tRNA synthetase (PDB id 1JIJ)**	CID446499	-681.22	-11.473	-13390.2
	**5k**	-678.81	-9.268	-13390.3
	CID446497	-677.09	-10.037	-13337.3
	CID446500	-676.65	-9.725	-13334.9
	CID446498 (cocryst. lig.)	-676.10	-9.552	-13327.4
**FtsZ (PDB id 4DXD)**	CID90135655	-696.62	-11.814	-13696.0
	CID25016417 (cocryst. lig.)	-696.44	-11.082	-13707.3
	**5k**	-693.60	-9.507	-13681.3
**Pyruvate kinase (PDB id 3T0T)**	CID10624851	-2476.14	-10.038	-49320.6
	**5k**	-2473.46	-9.572	-49277.2
	CID135642239 (cocryst. lig.)	-2473.28	-11.023	-49244.4
**Gyrase B (PDB id 3G7B)**	CID11718729	-445.73	-7.911	-8755.1
	CID44608008 (cocryst. lig.)	-445.63	-7.091	-8760.9
	CID2744426	-443.53	-6.929	-8723.8
	CID54759160	-443.33	-6.890	-8728.9
	**5k**	-442.92	-8.751	-8682.8
	CID153541355	-442.53	-7.322	-8704.1
**CrtM (PDB id 2ZCQ)**	CID56928041	-688.91	-13.583	-13506.6
	CID44182294	-688.52	-12.589	-13515.1
	CID24748047 (cocryst. lig.)	-686.69	-12.236	-13485.2
	CID56928042	-686.06	-11.770	-13485.8
	**5k**	-682.43	-10.927	-13429.6
**Thymidylate kinase TMK (PDB id 4HLC)**	CID66545765	-455.97	-10.406	-8910.8
	CID66553127 (cocryst. lig.)	-455.95	-10.843	-8902.2
	CID66553128	-453.73	-8.513	-8898.4
	**5k**	-449.39	-8.185	-8823.6

**Dihydrofolate reductase DHFR (PDB id 3FYW)**	CID10247560	-375.62	-9.797	-7306.7
	CID53346505	-373.68	-9.799	-7276.4
	CID42627761	-373.16	-8.619	-7281.2
	CID5578	-373.07	-8.021	-7291.2
	**5k**	-364.46	-7.644	-7135.7

Supporting Figure S1. 3D representations of the best docked poses of the co-crystallized inhibitors for tyrosyl-tRNA synthetase (panel a), Gyrase B (panel b), and Pyruvate Kinase (panel c) from *S. aureus* in complex with their respective target proteins.

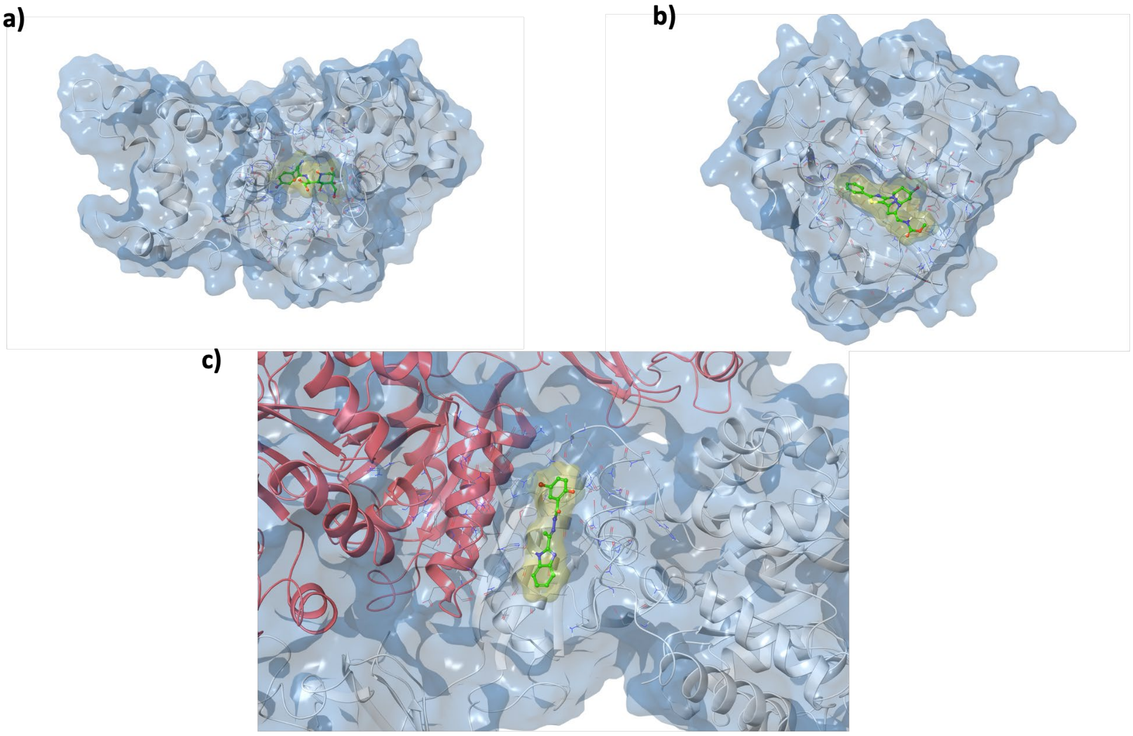

Supporting Figure S2. Protein backbone and ligand RMSD (Å) plots for the trajectories of the reference co-resolved inhibitors of tyrosyl-tRNA synthetase (panel a), Gyrase B (panel b), and Pyruvate Kinase (panel c) from *S. aureus* in complex with the correspondent target proteins over a 100 ns timescale.

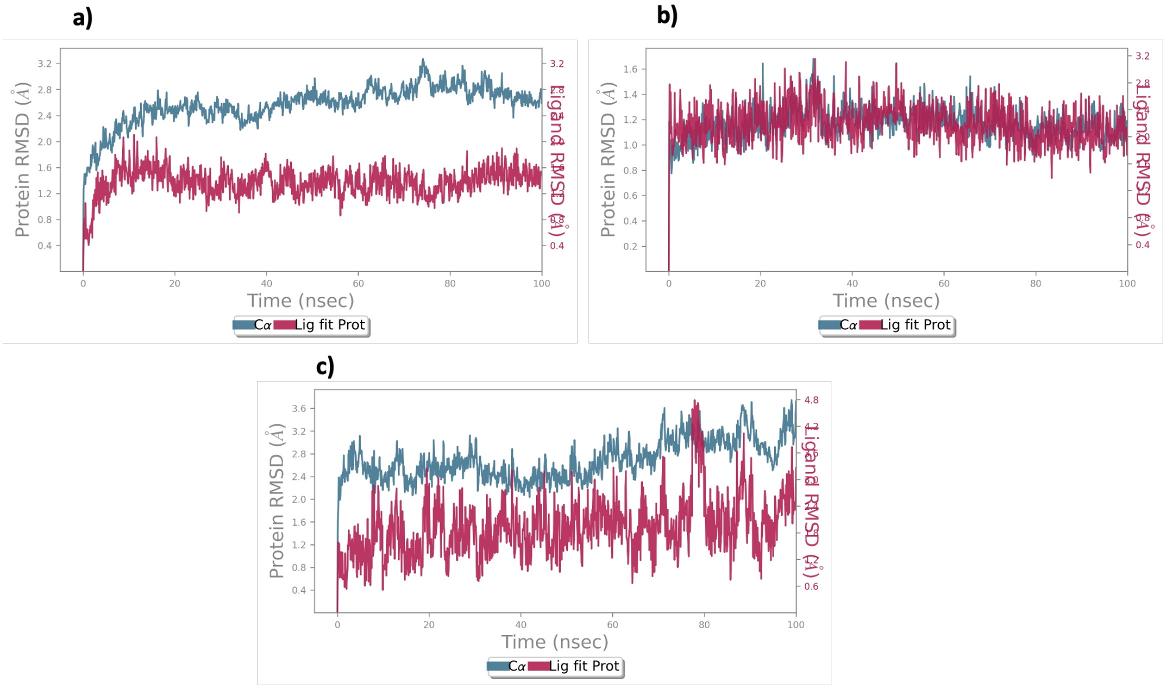

S3. Supporting material for in vitro assays on *S. aureus*

Supporting Figure S3. Dose-dependent effects of **5k** against *S. aureus* ATCC 25923 cell viability. Minimal Inhibitory Concentration and the Minimal Bactericidal Concentration of the **5k** novel 6,7-dimethoxy-4-piperazinyl quinoline derivative against the reference pathogen *S. aureus* ATCC25923 .

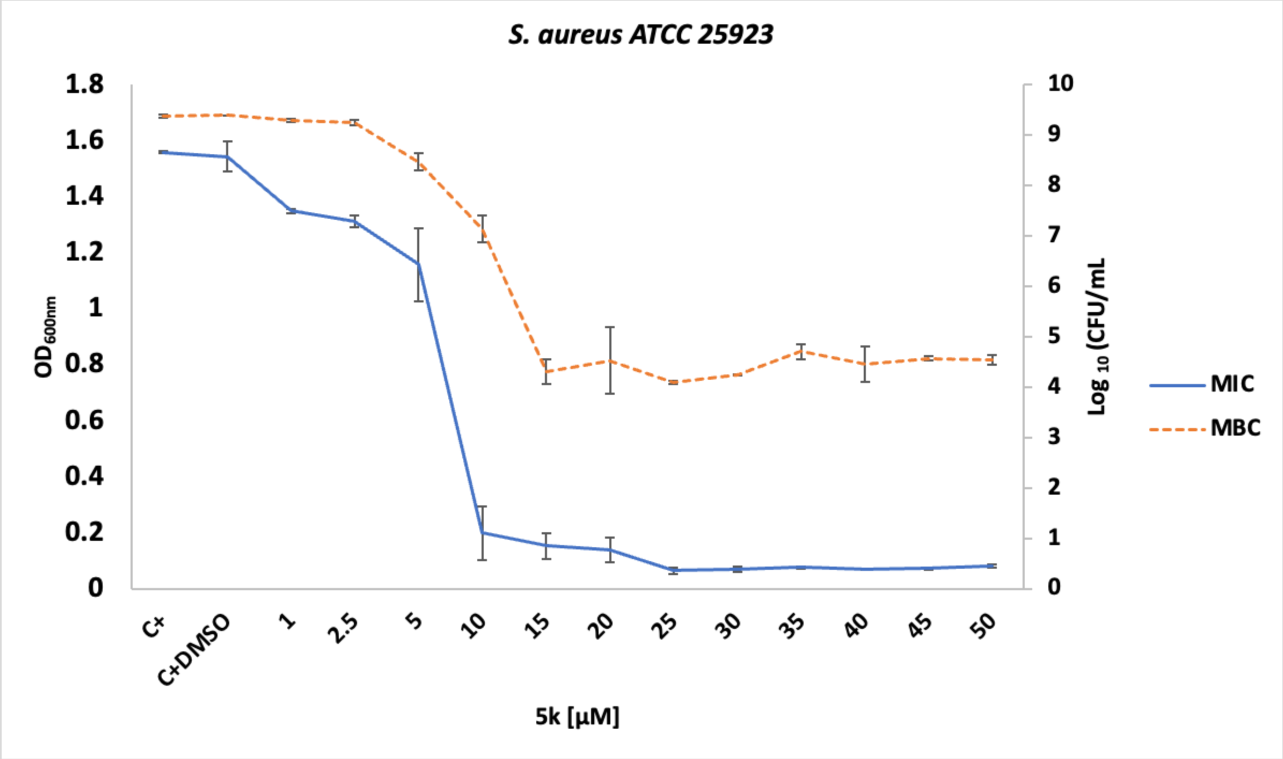

Supporting Figure S4. Dose-dependent effects of **5k** against *S. aureus* ATCC 25923 cell viability. Minimal Inhibitory Concentration and the Minimal Bactericidal Concentration of the **5k** against the reference pathogen *S. aureus* ATCC25923.

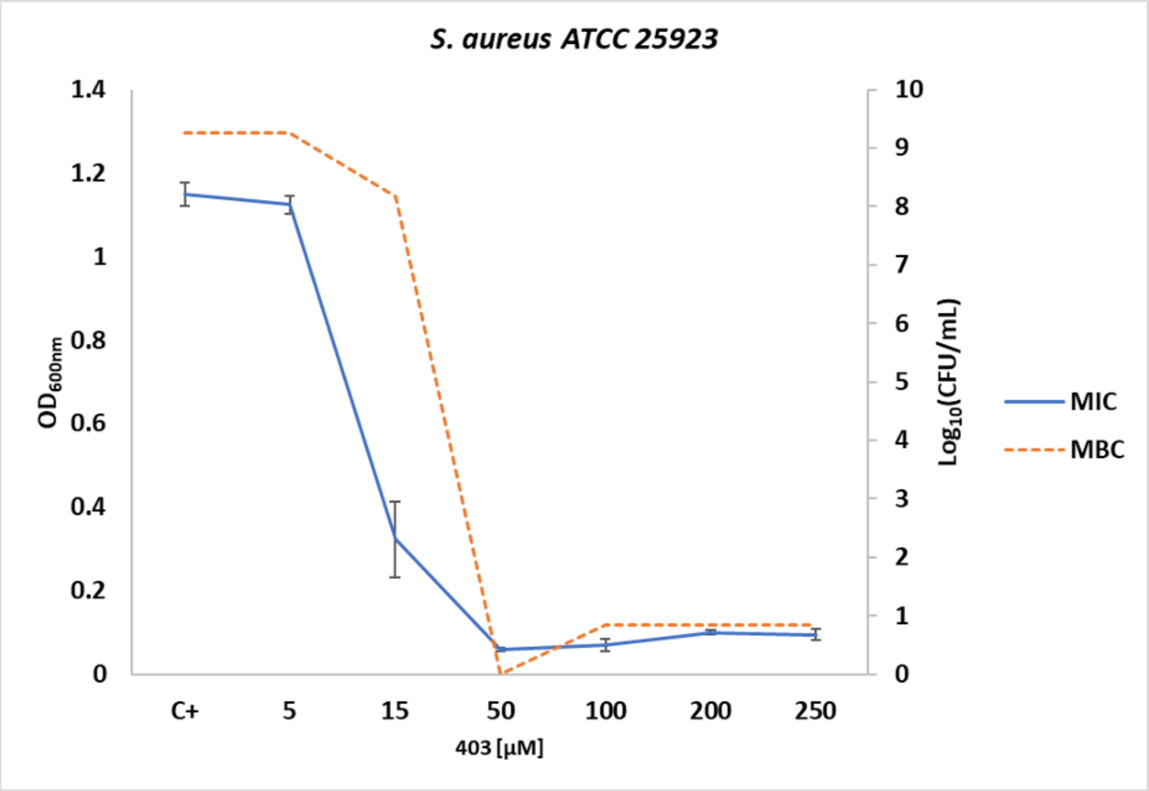

S4. ^{1}H and ^{13}C NMR spectra for compounds 10 and 5a-k

^{1}HNMR compound **10** (400 MHz, $CDCl_3$)

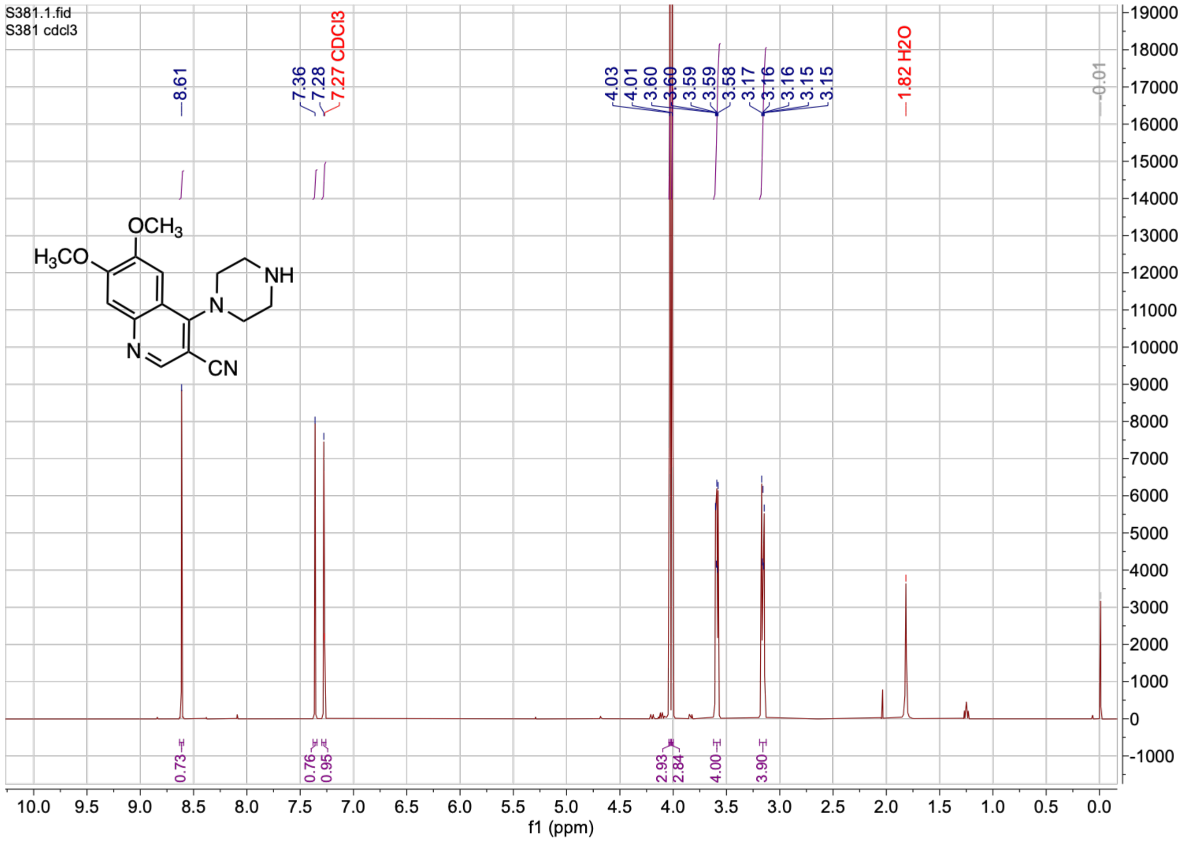

^{13}CNMR compound **10** (100 MHz, $CDCl_3$)

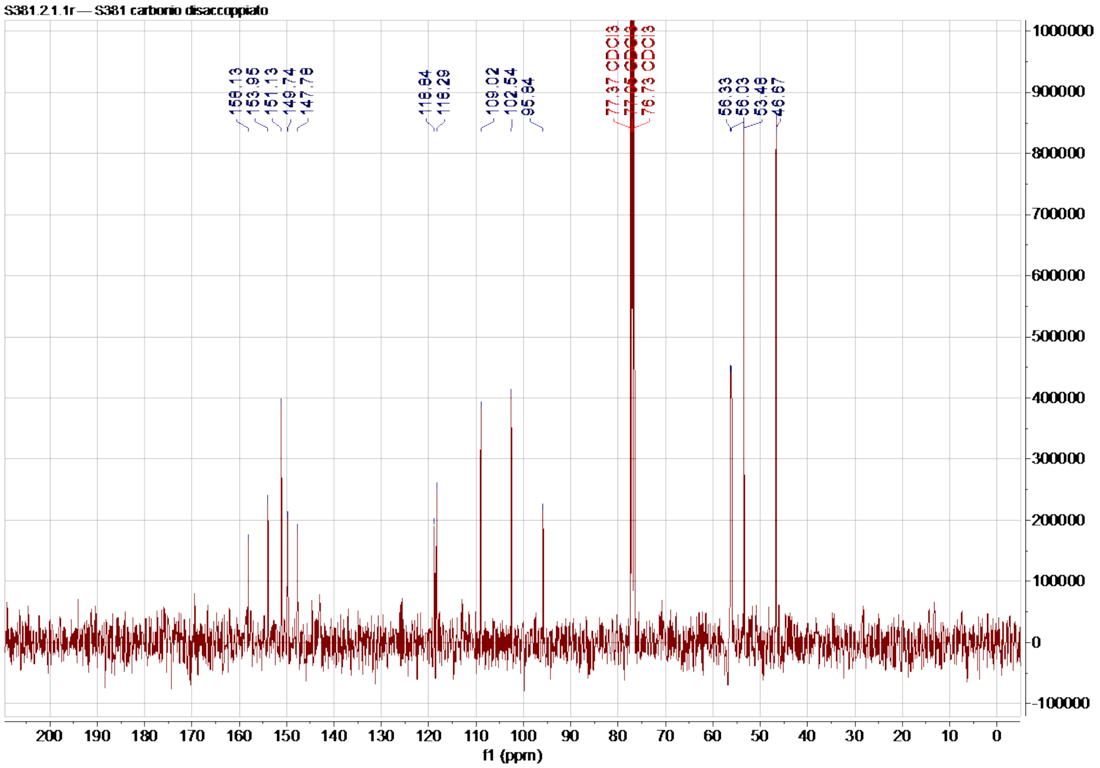

^{1}HNMR compound **5a** (400 MHz, $CDCl_3$)

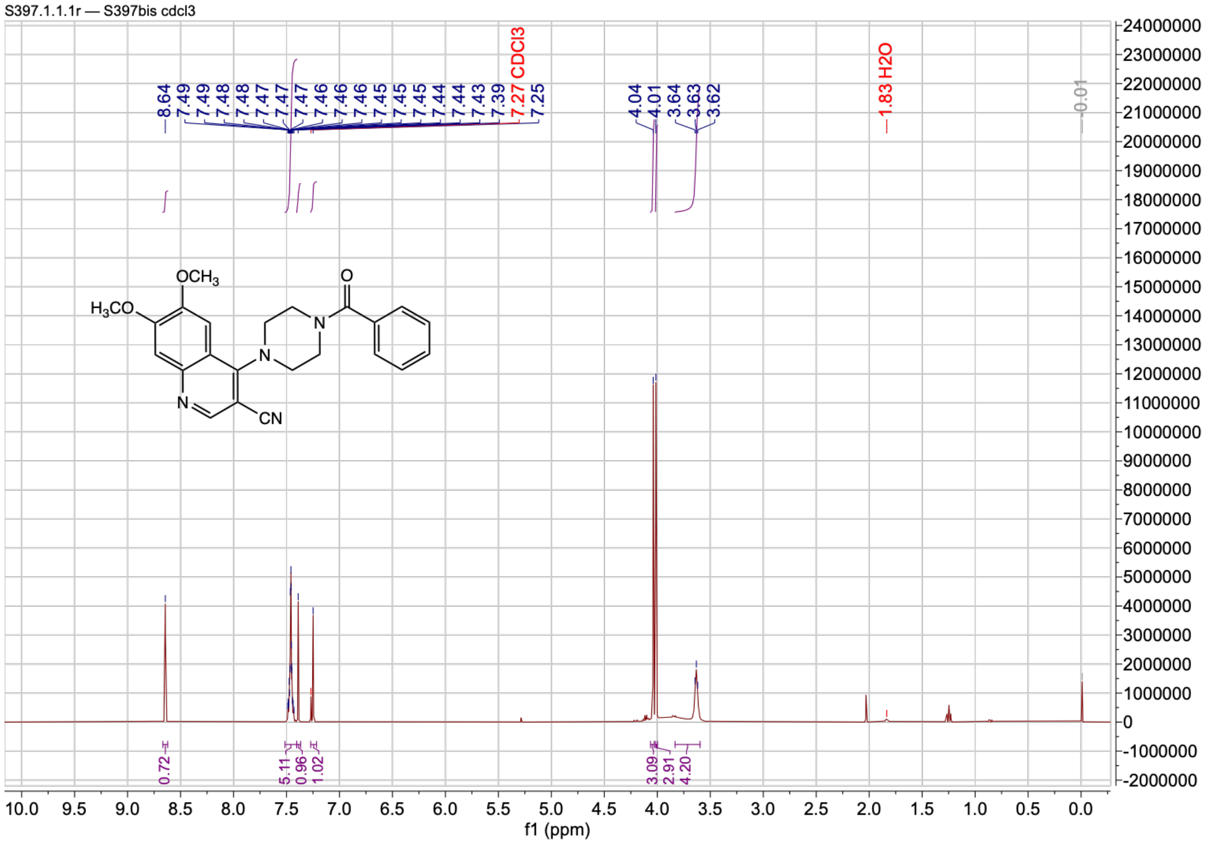

^{13}CNMR compound **5a** (100 MHz, $CDCl_3$)

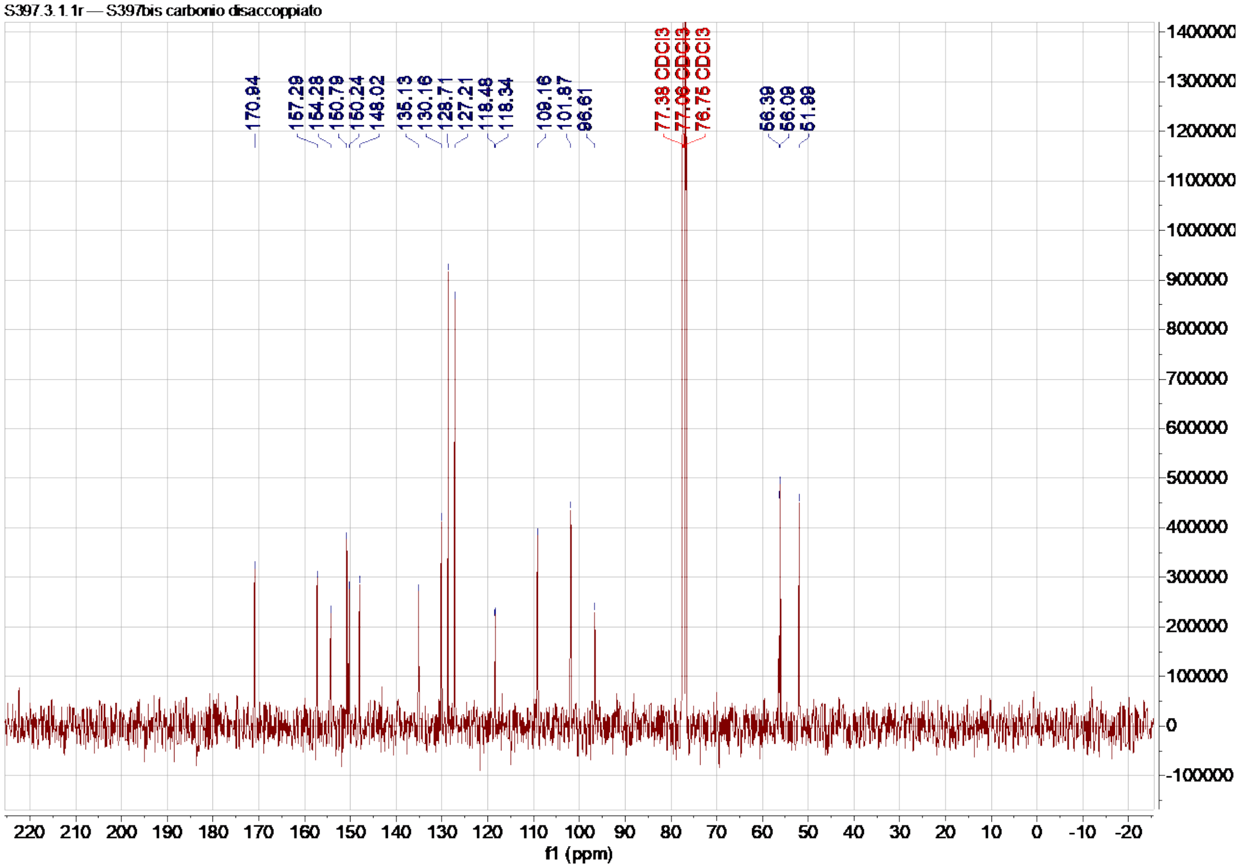

^{1}HNMR compound **5b** (400 MHz, DMSO-d_6)

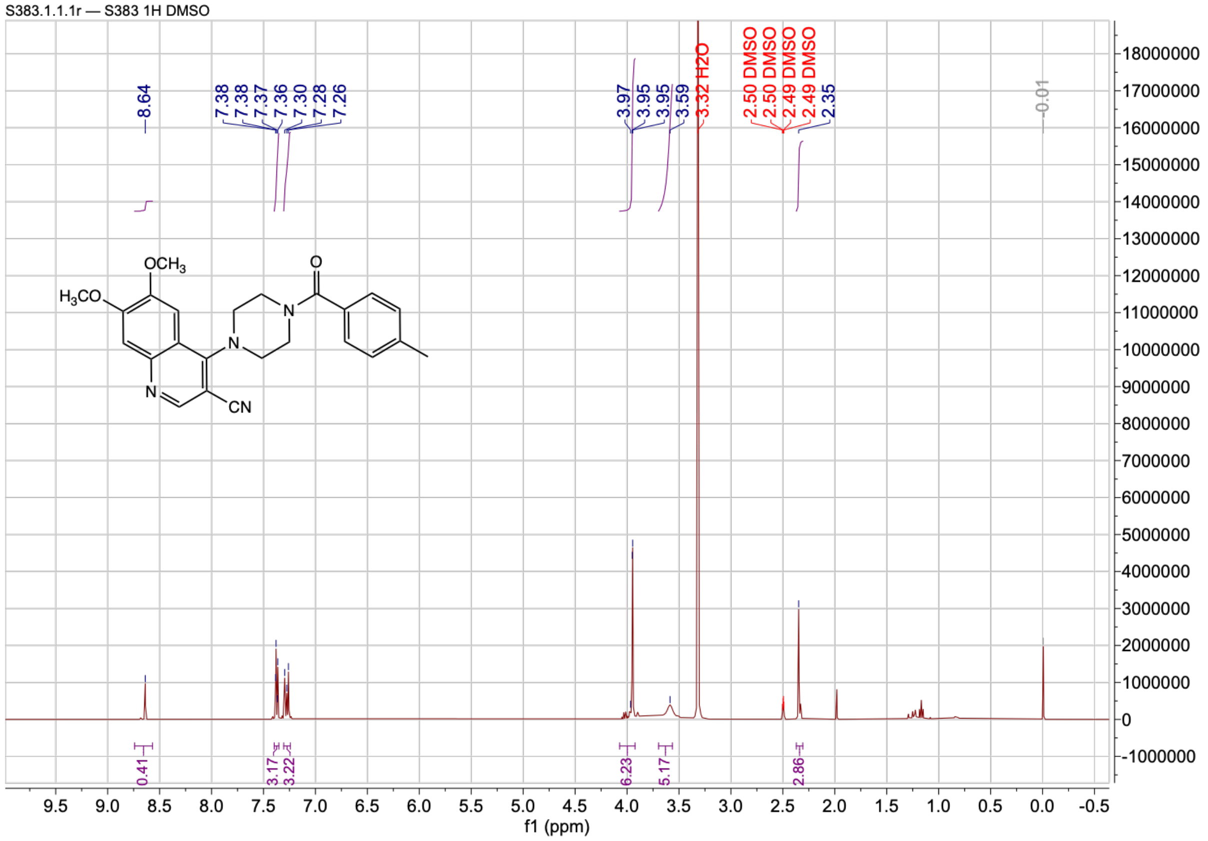

^{13}CNMR compound **5b** (100 MHz, DMSO-d_6)

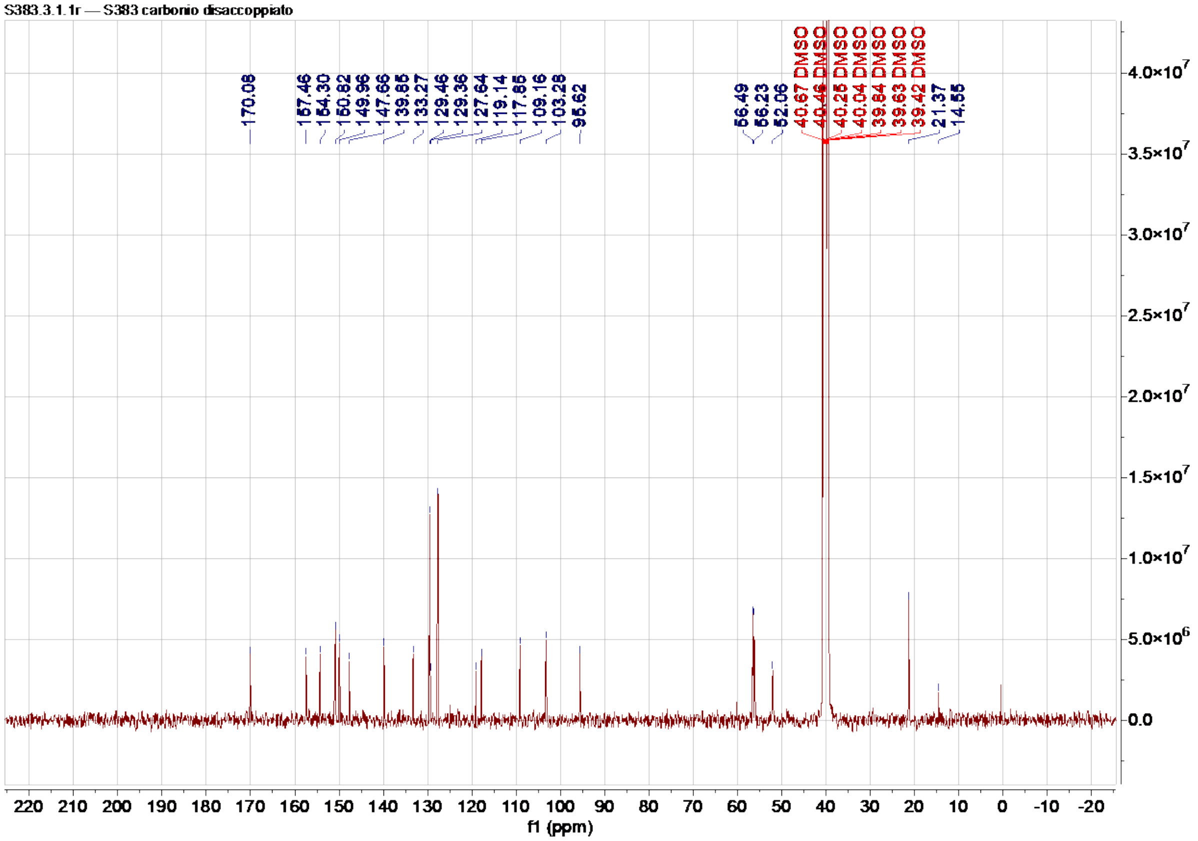

^{1}HNMR compound **5c** (400 MHz, $CDCl_3$)

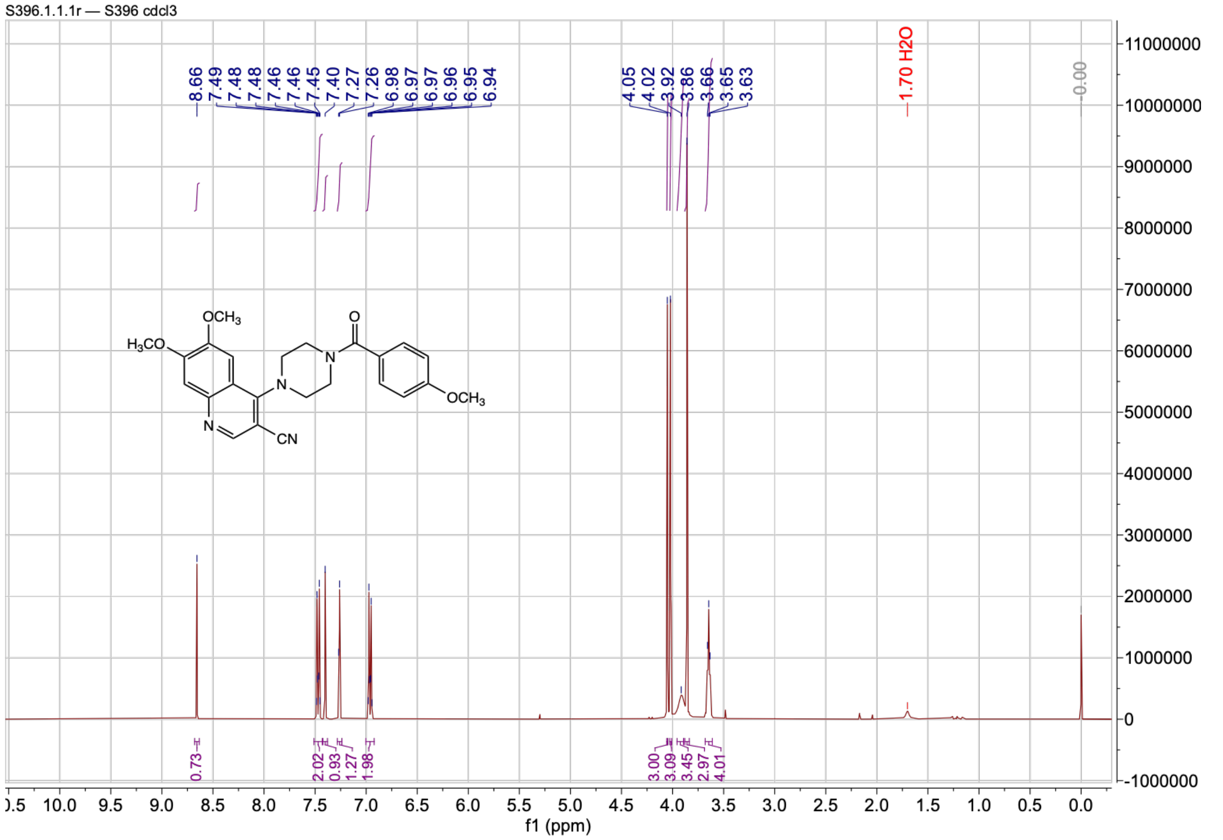

^{13}CNMR compound **5c** (100 MHz, $CDCl_3$)

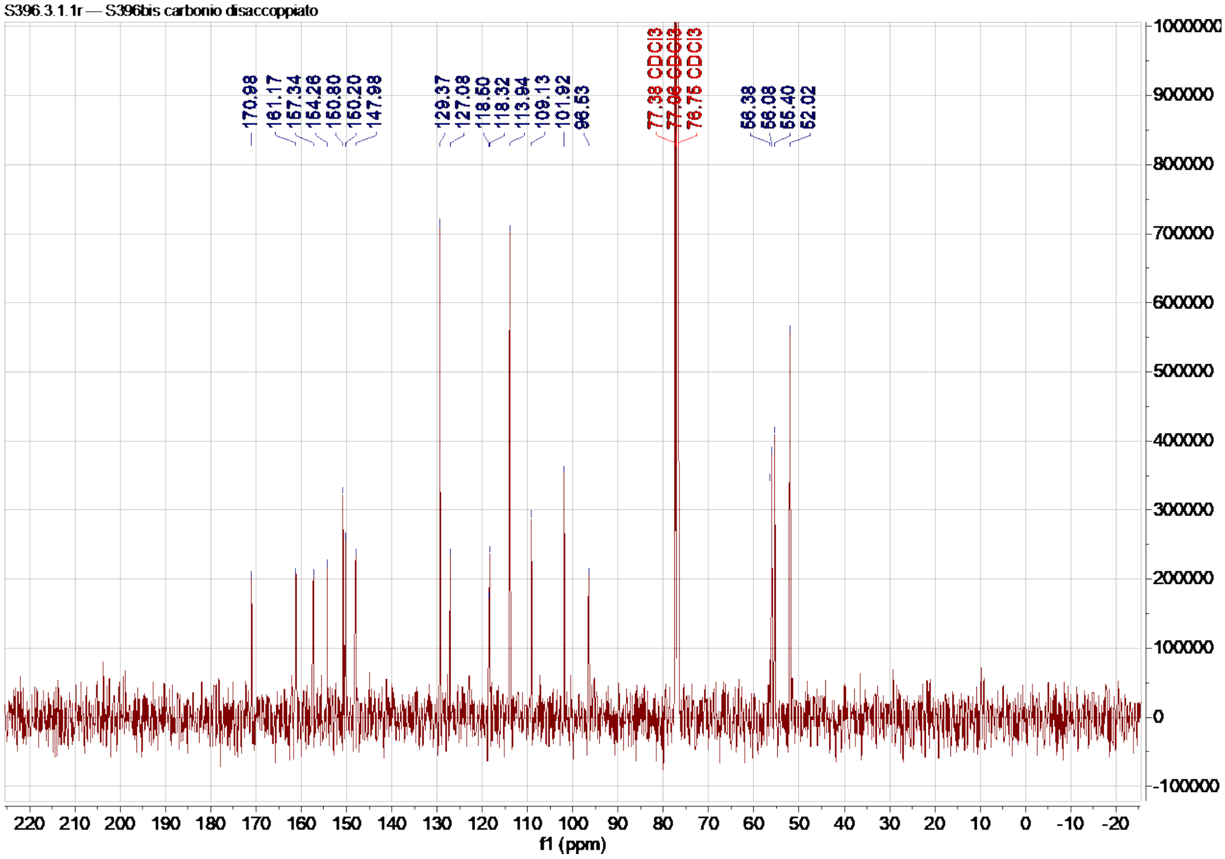

^{1}HNMR compound **5d** (400 MHz, $CDCl_3$)

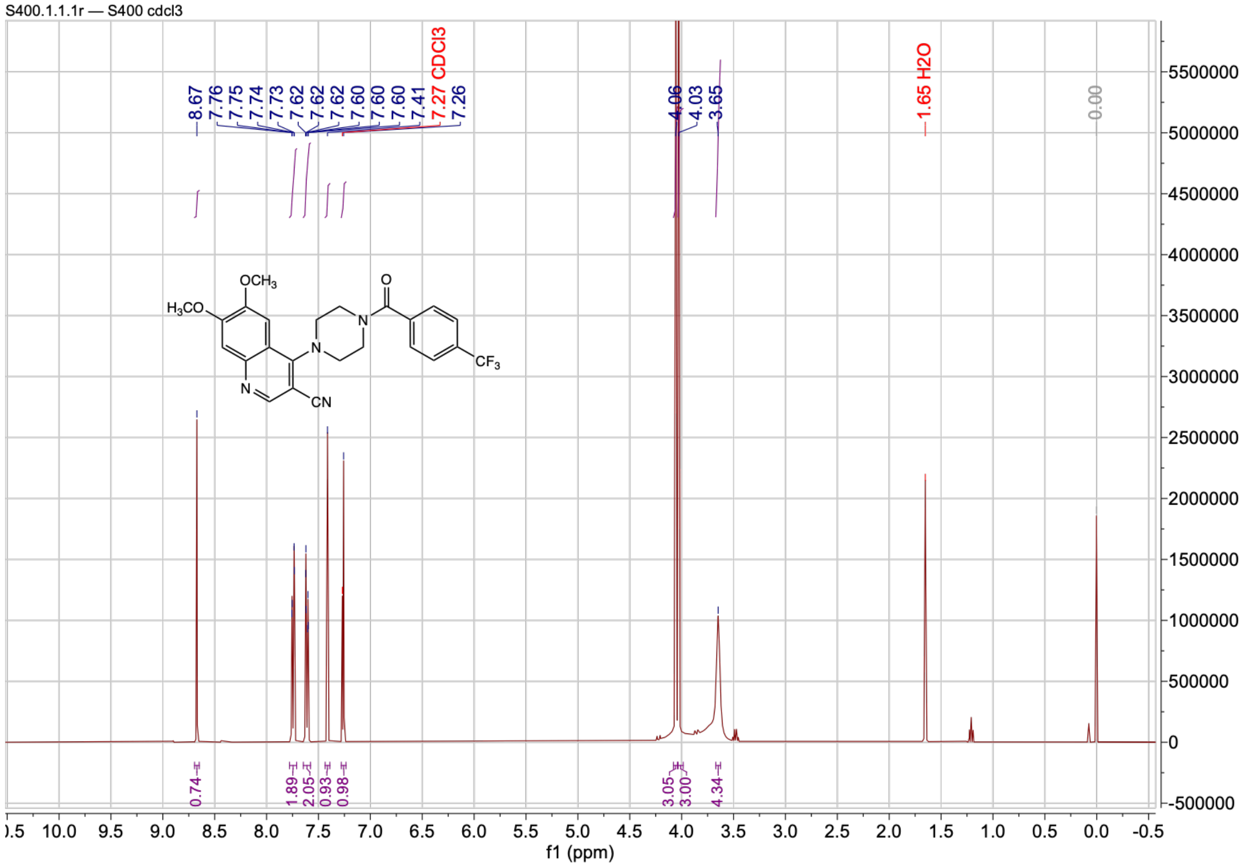

^{13}CNMR compound **5d** (100 MHz, $CDCl_3$)

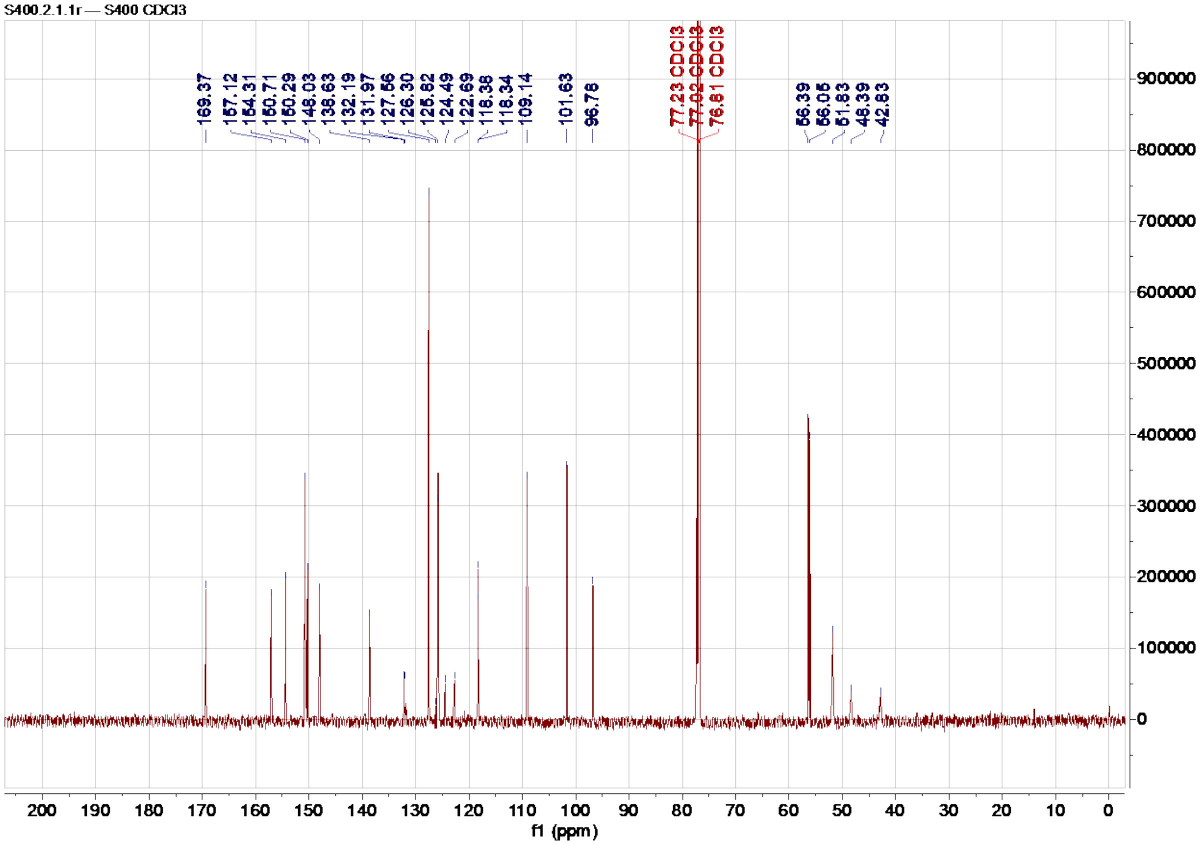

^{1}HNMR compound **5e** (400 MHz, $CDCl_3$)

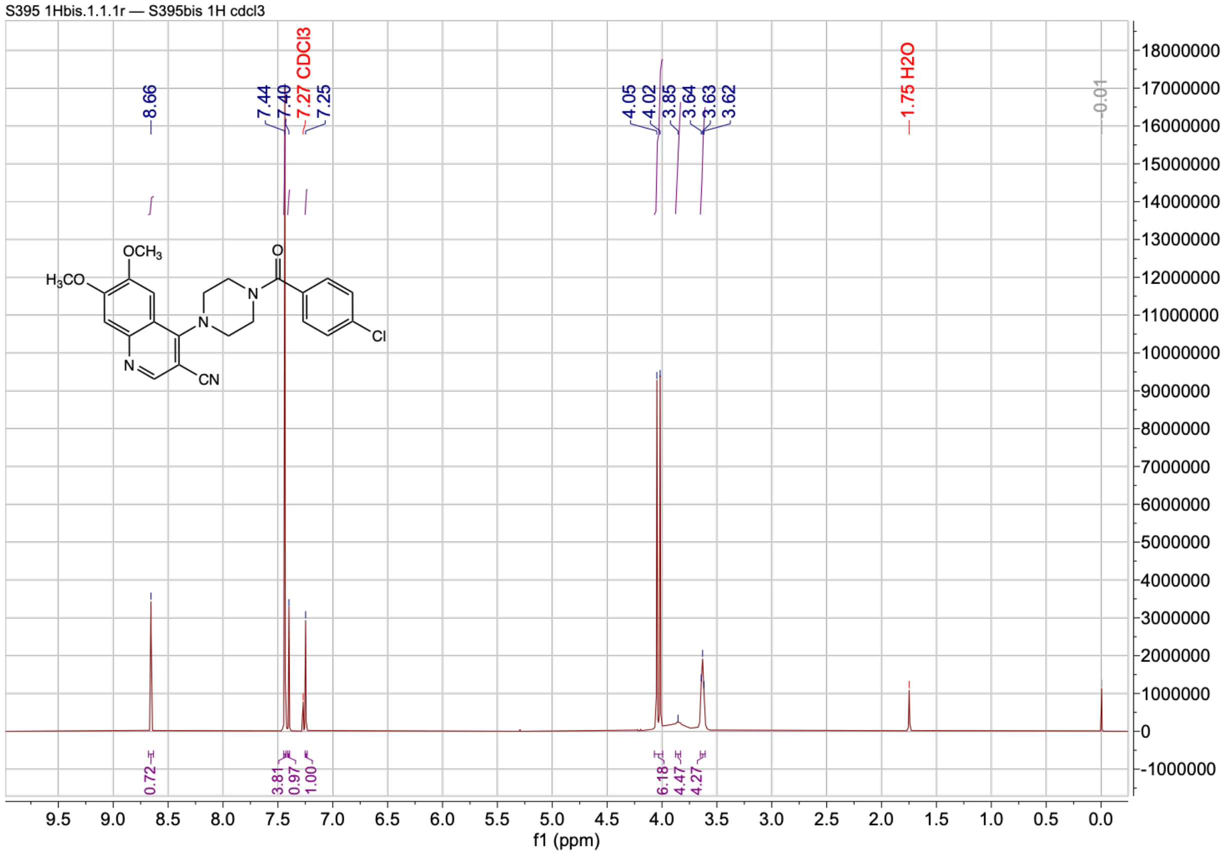

^{13}CNMR compound **5e** (100 MHz, $CDCl_3$)

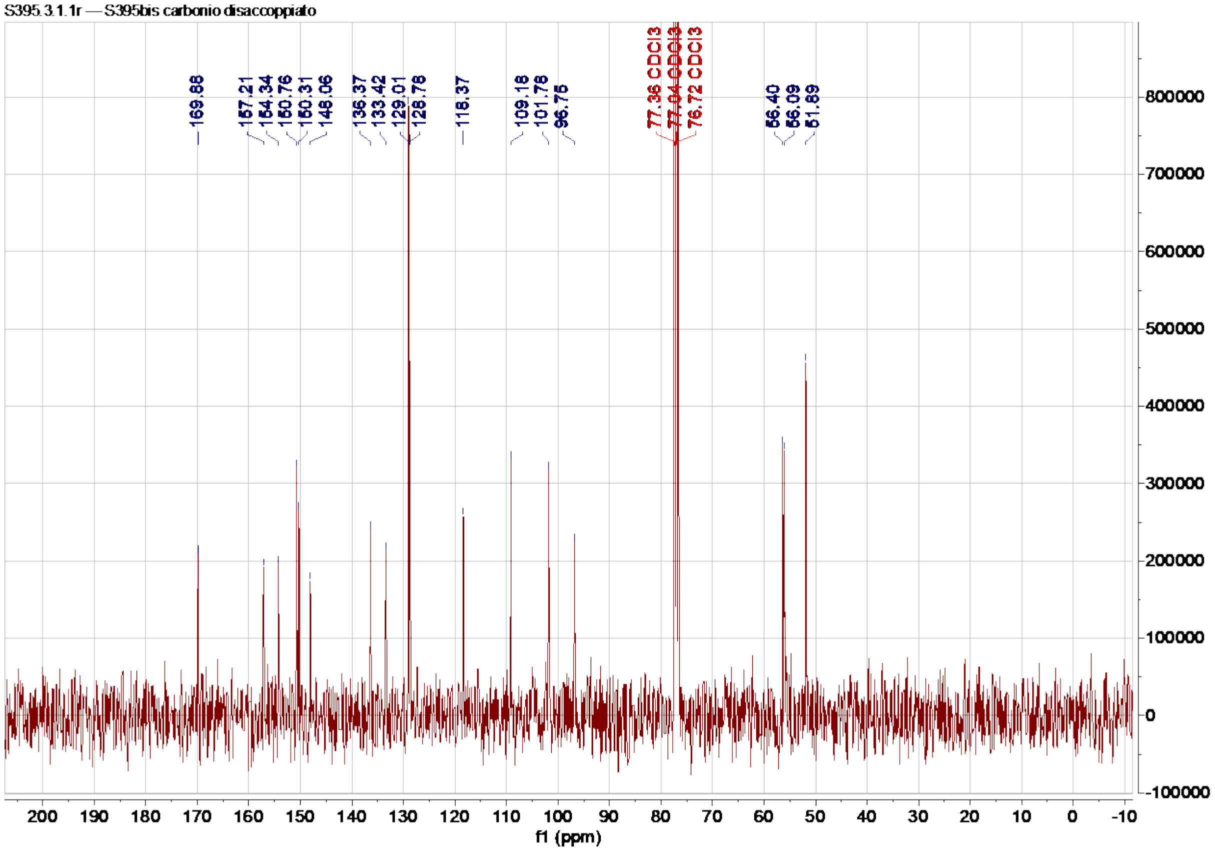

^{1}HNMR compound **5f** (400 MHz, $CDCl_3$)

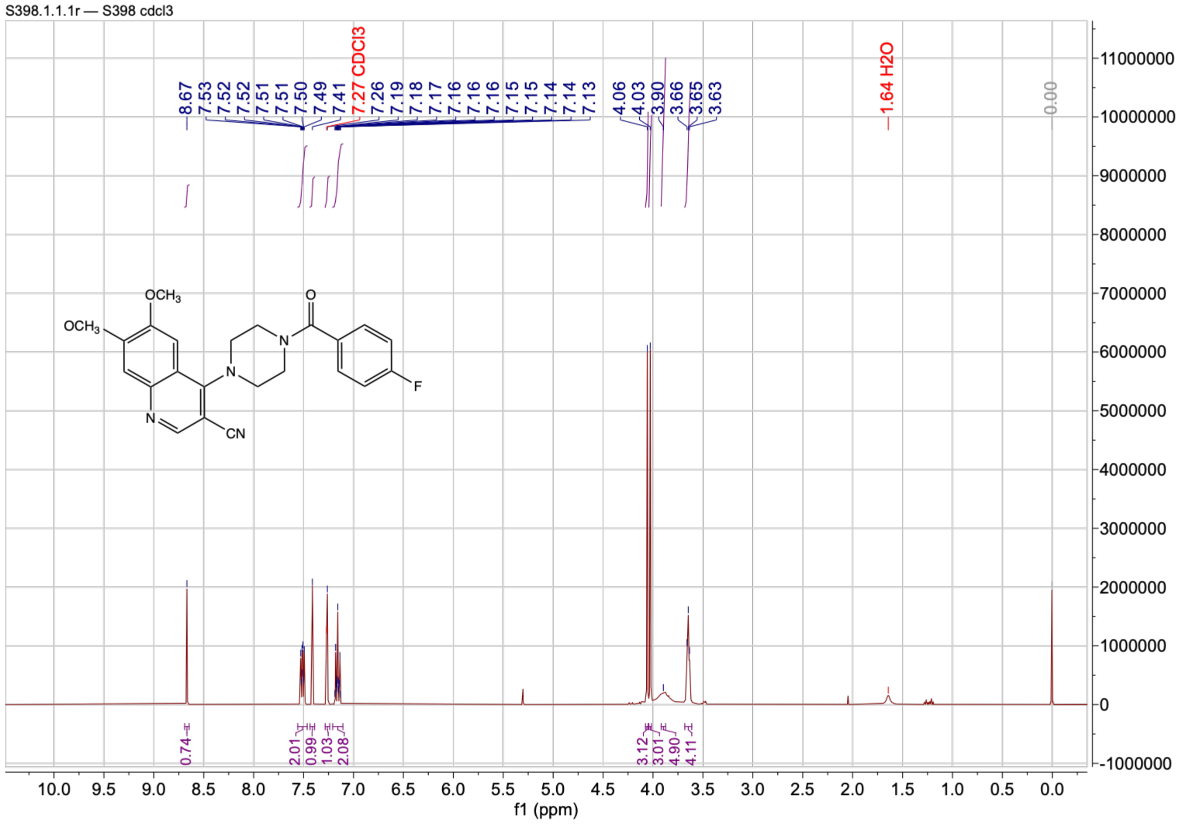

^{13}CNMR compound **5f** (100 MHz, $CDCl_3$)

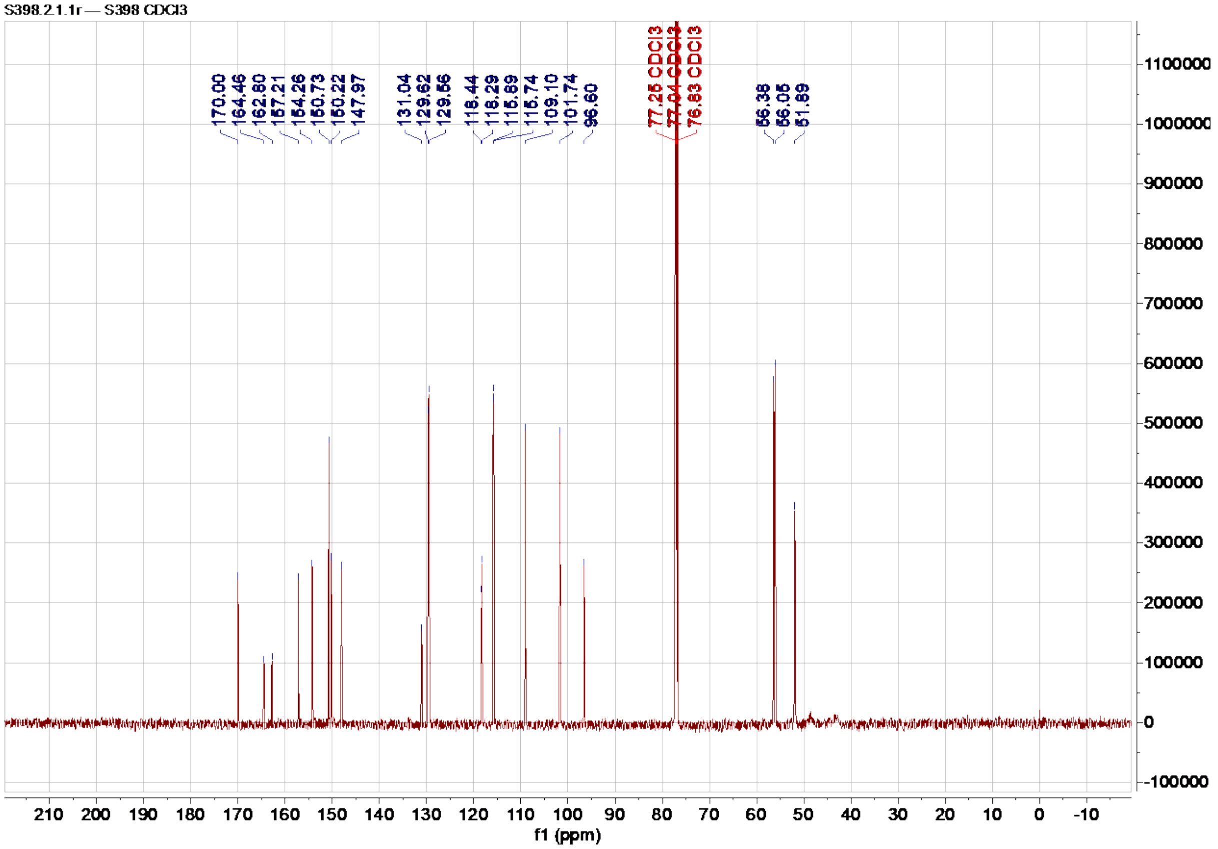

^{1}HNMR compound **5g** (400 MHz, $CDCl_3$)

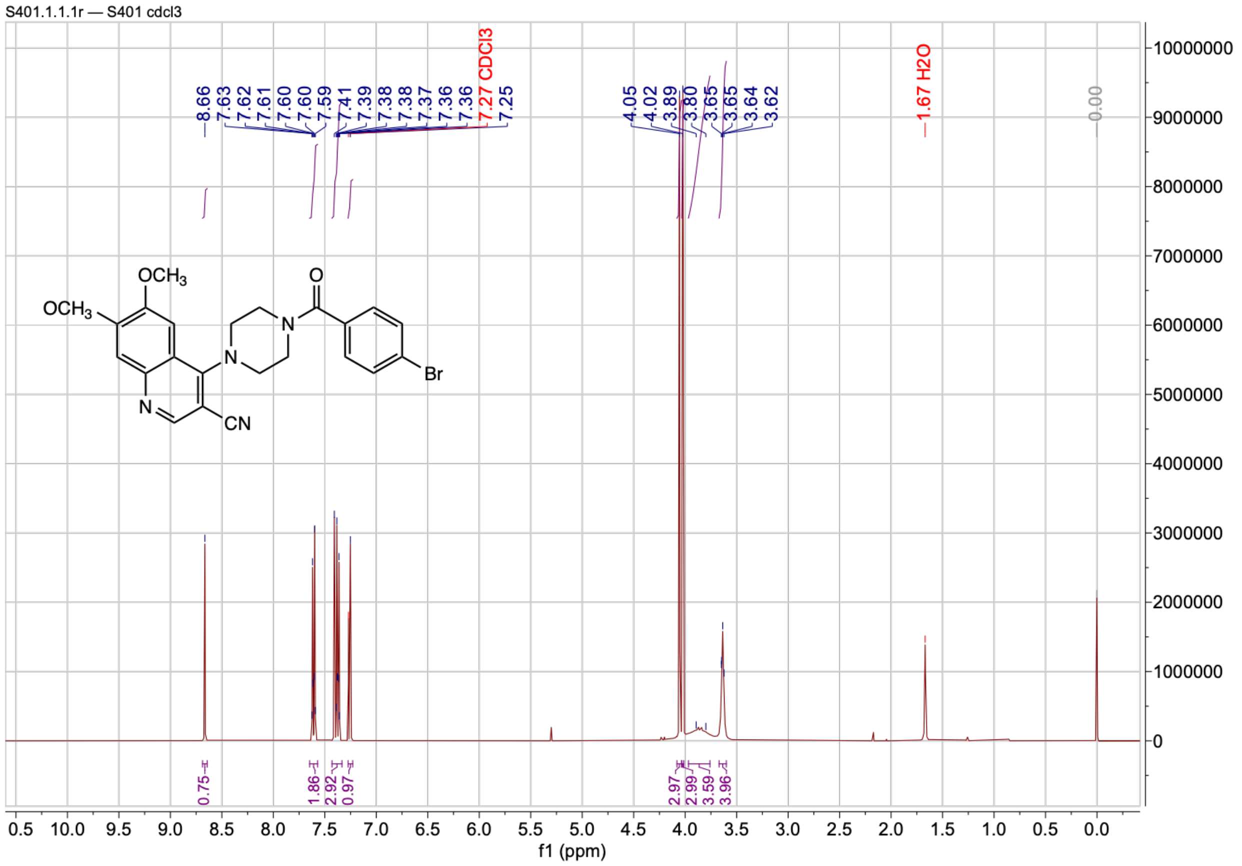

^{13}CNMR compound **5g** (100 MHz, $CDCl_3$)

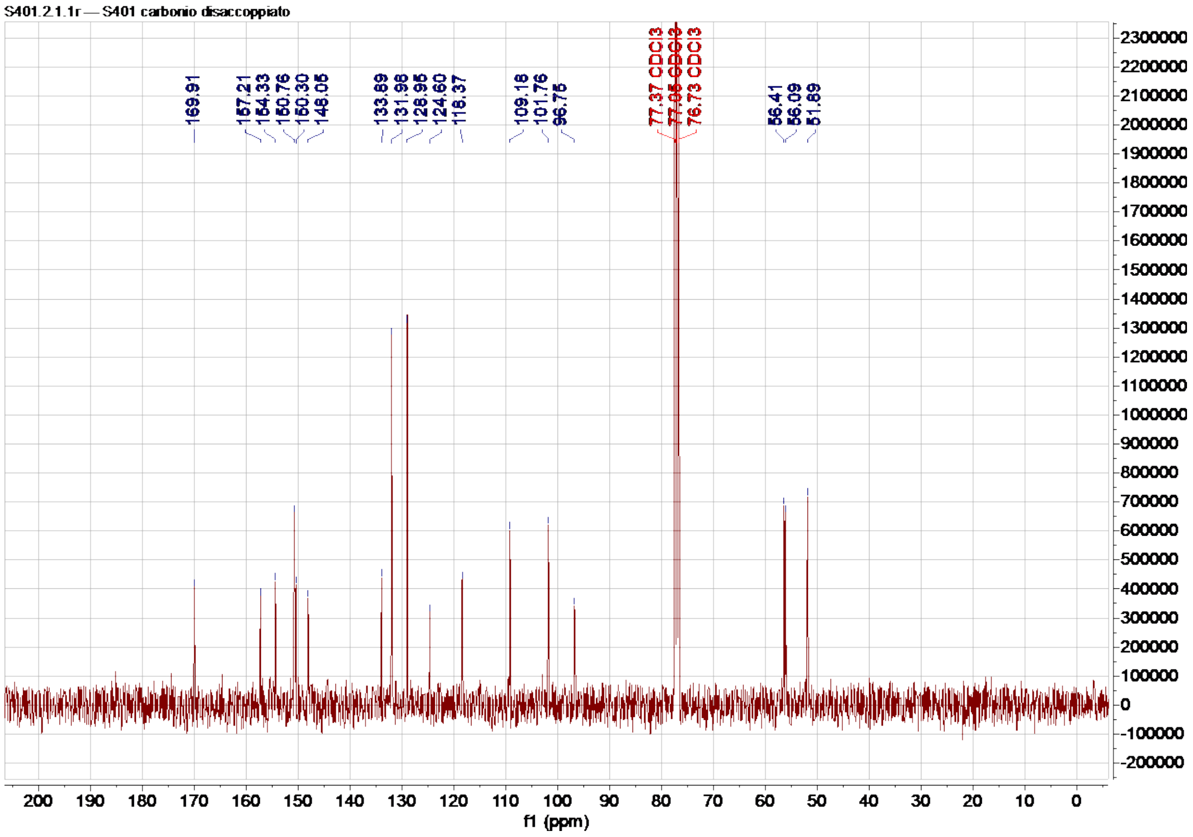

^{1}HNMR compound **5h** (400 MHz, $CDCl_3$)

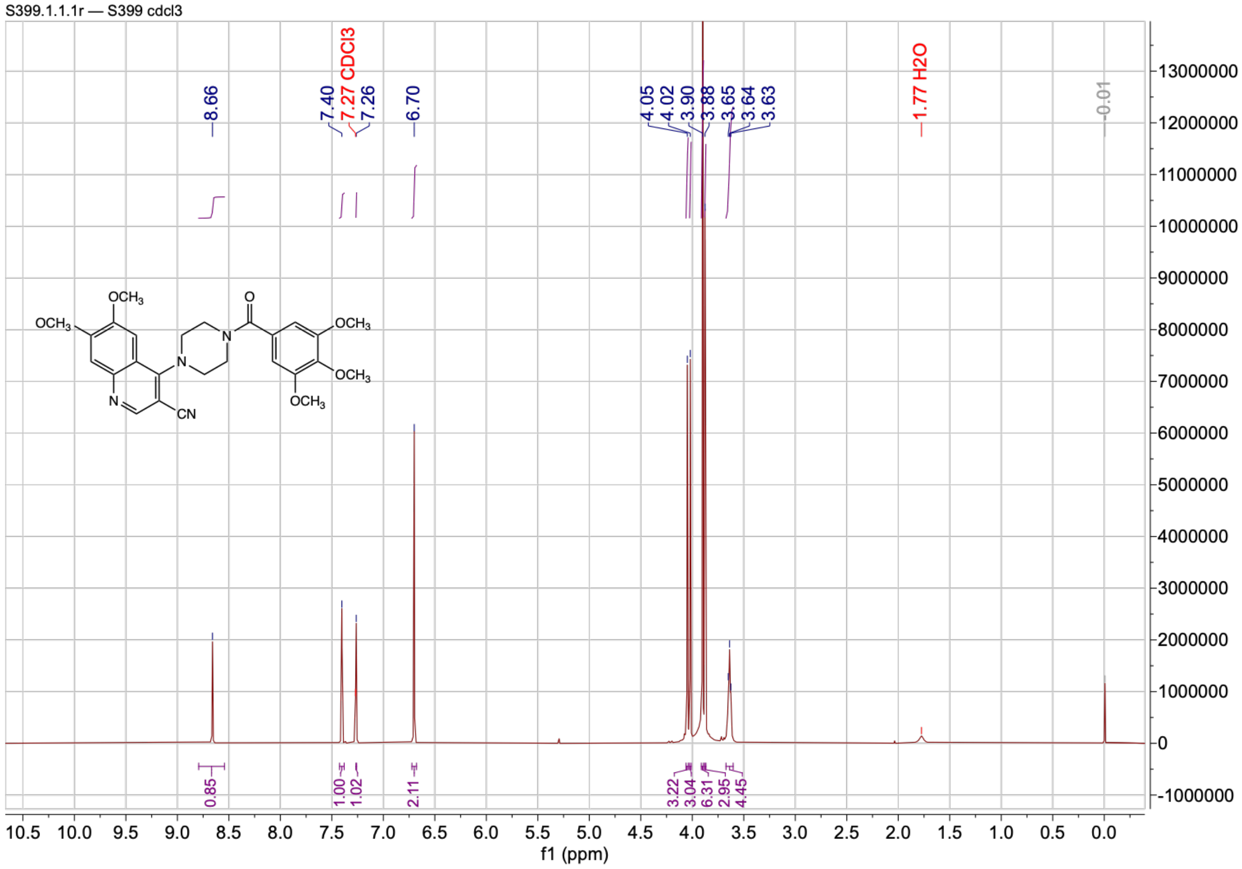

^{13}CNMR compound **5h** (100 MHz, $CDCl_3$)

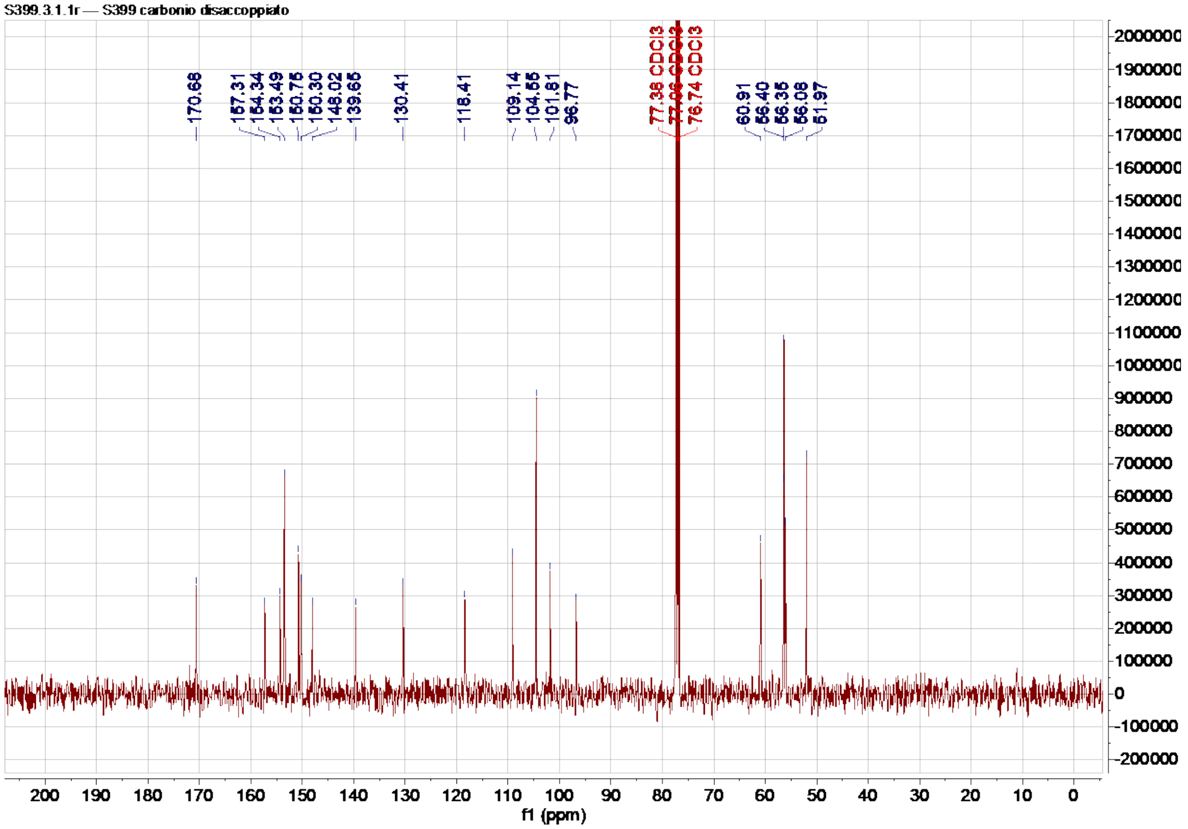

^{1}HNMR compound **5i** (400 MHz, $CDCl_3$)

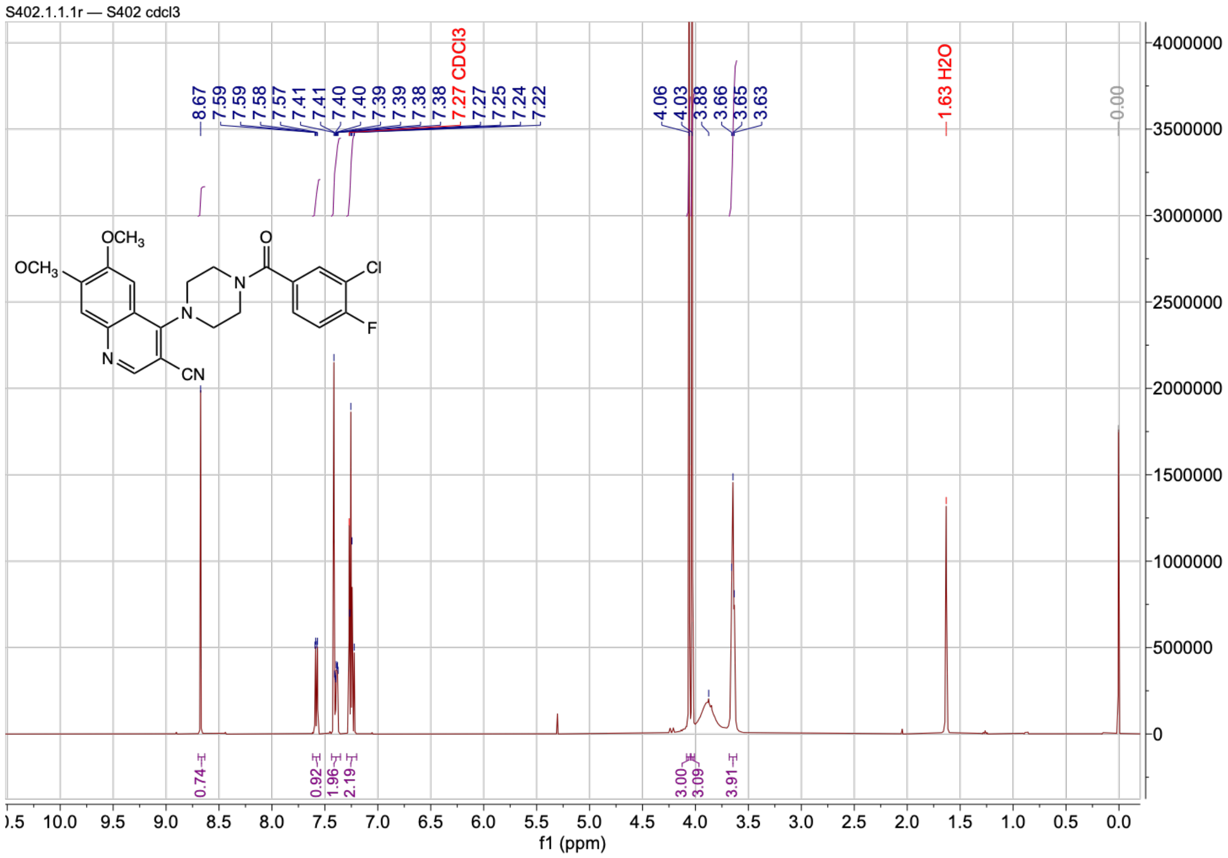

^{13}CNMR compound **5i** (100 MHz, $CDCl_3$)

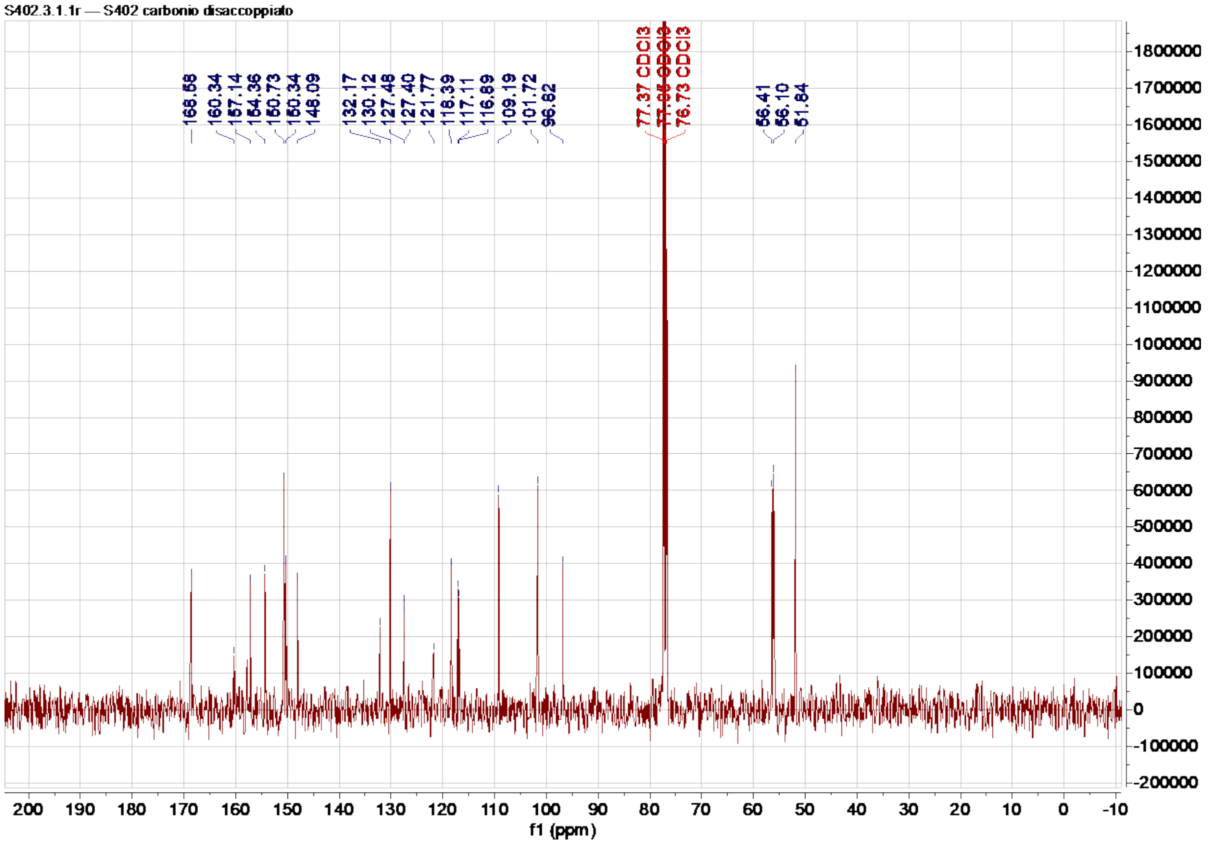

^{1}HNMR compound **5j** (400 MHz, $CDCl_3$)

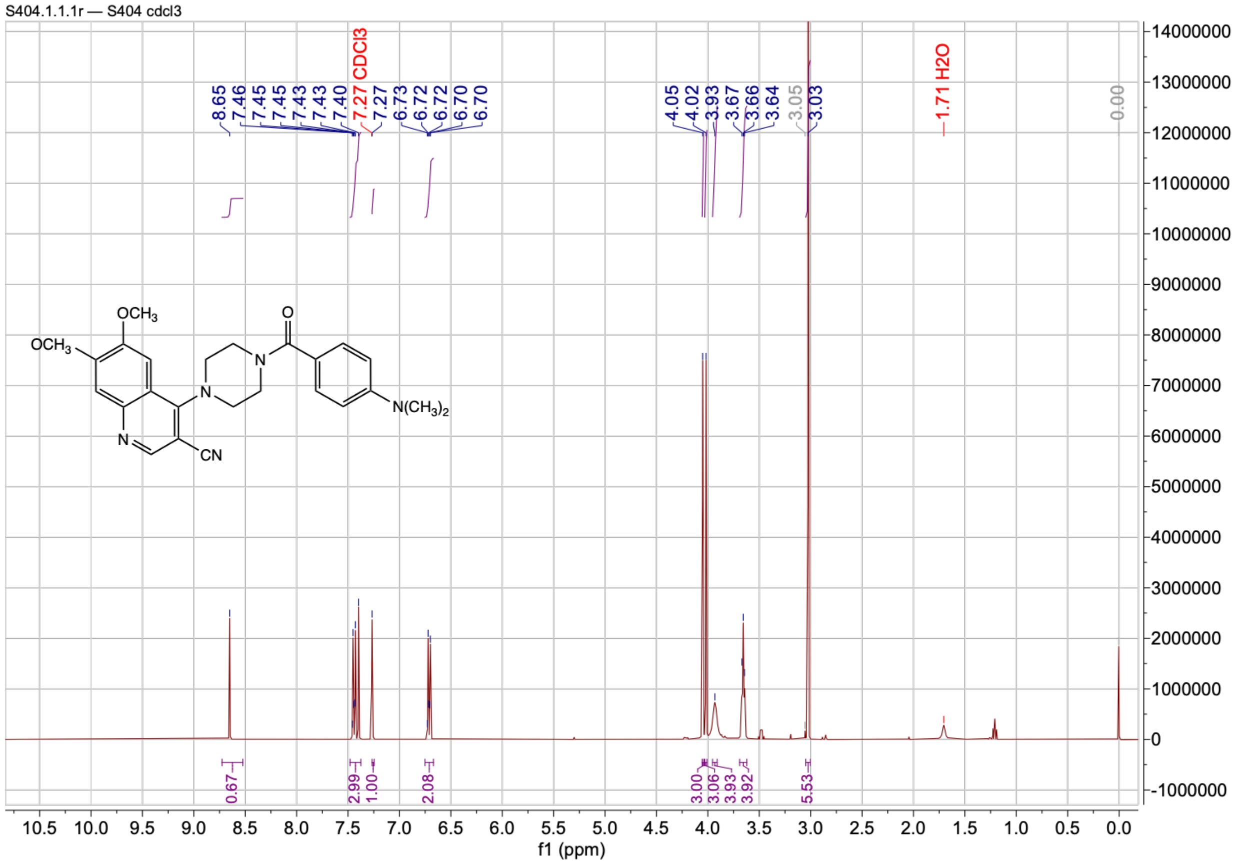

^{13}CNMR compound **5j** (100 MHz, $CDCl_3$)

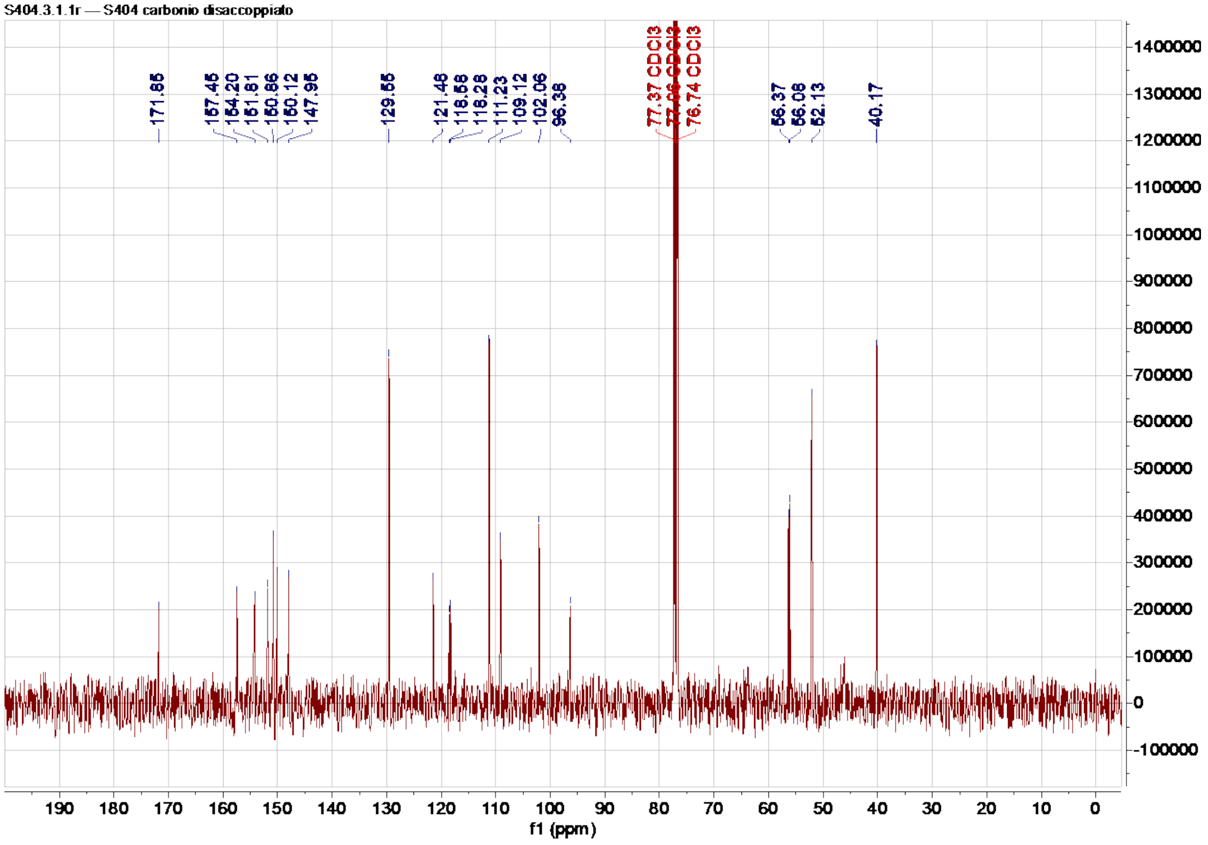

^{1}HNMR compound **5k** (400 MHz, $CDCl_3$)

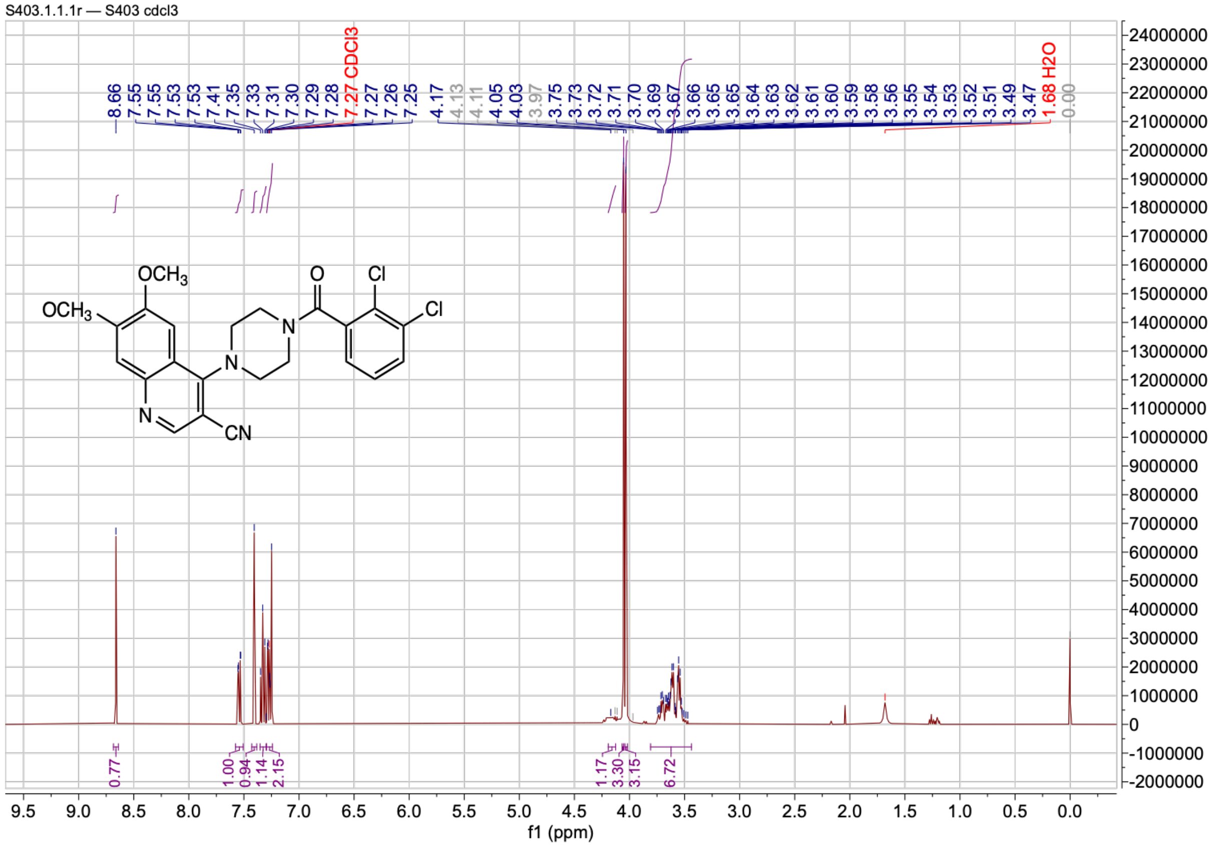

^{13}CNMR compound **5k** (100 MHz, $CDCl_3$)

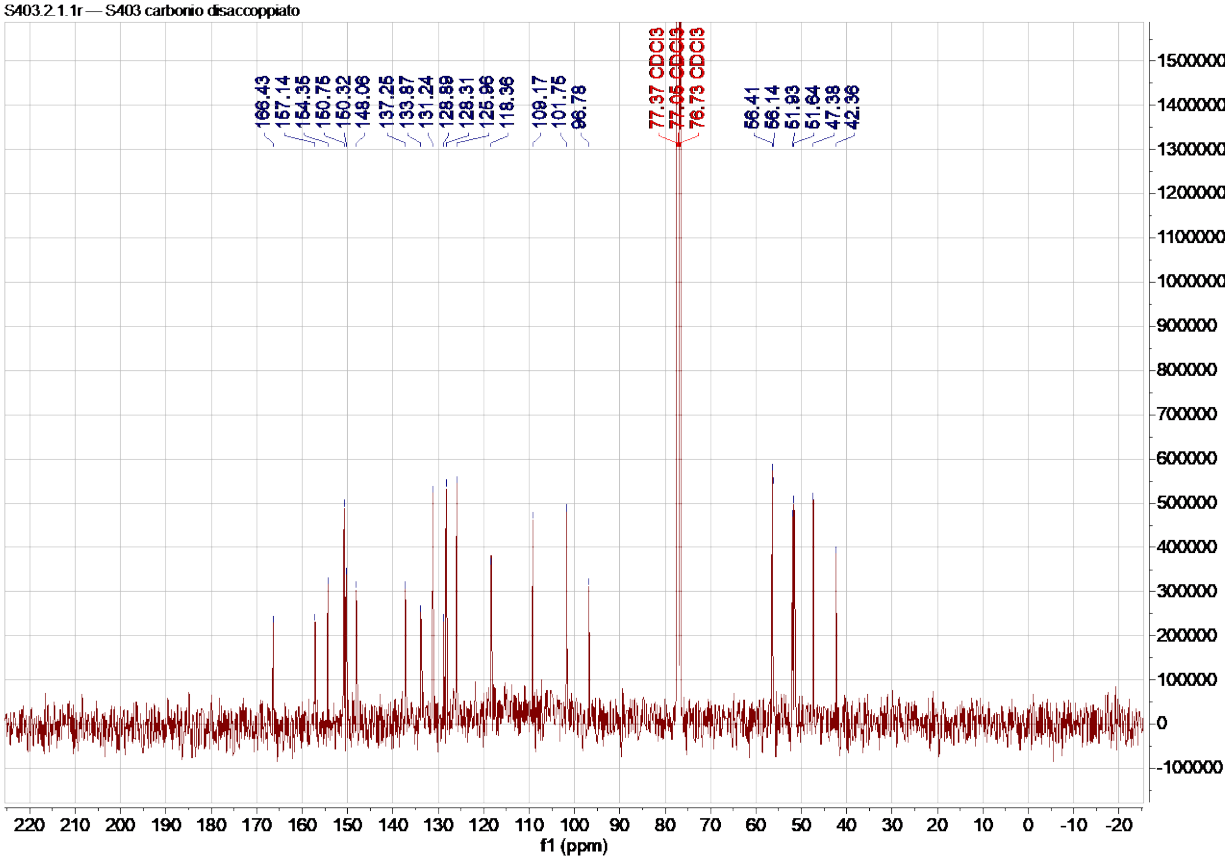

Supplement: Supplementary file 1 [file molecules-30-00028-s001.zip › Supporting information S2-4_8 novembre revised.pdf]
